# Epigenetic changes and alternate promoter usage by human colon cancers for expressing DCLK1-isoforms: Clinical Implications

**DOI:** 10.1038/srep14983

**Published:** 2015-10-08

**Authors:** Malaney R. O’Connell, Shubhashish Sarkar, Gurinder K. Luthra, Yoshinaga Okugawa, Yuji Toiyama, Aakash H. Gajjar, Suimin Qiu, Ajay Goel, Pomila Singh

**Affiliations:** 1 Departments of Neuroscience and Cell Biology, UTMB, Galveston, TX; 2 Gastroenterology Division in Internal Medicine, UTMB, Galveston, TX; 3 Gastrointestinal Cancer Research Laboratory, Division of Gastroenterology, Department of Internal Medicine, Charles A. Sammons Cancer Center and Baylor Research Institute, Baylor University Medical Center, Dallas, Texas, USA; 4 Department of Gastrointestinal and Pediatric Surgery, Division of Reparative Medicine, Institute of Life Sciences, Graduate School of Medicine, Mie University, Mie 514-8507, Japan; 5 Surgery, UTMB, Galveston, TX; 6 Pathology, UTMB, Galveston, TX.

## Abstract

DCLK1 specifically marks colon/pancreatic cancers in mice, and is expressed by human colon adenocarcinomas (hCRCs). Down-regulation of DCLK1 results in loss of cancer-stem-cells (CSCs), and inhibits spheroidal/xenograft growths from hCRC-cells. The 5′-promoter of DCLK1-gene is reportedly hypermethylated in hCRCs, resulting in loss of expression of DCLK1-transcripts, originating from 5′(α)-promoter (termed DCLK1-L, in here). However, in mouse colon-tumors, 5′-promoter of DCLK1-gene remains unchanged, and DCLK1-L, originating from 5′(α)-promoter, is expressed. We hypothesized that elevated levels of DCLK1-protein in hCRC-cells, may be transcribed/translated from an alternate-promoter. Several in silico and molecular biology approaches were used to test our hypothesis. We report for the first time that majority of hCRCs express short-transcripts of DCLK1 (termed DCLK1-S, in here) from an alternate β-promoter in IntronV of the gene, while normal-colons mainly express DCLK1-L from 5′(α)-promoter. We additionally report an important role of β-catenin and TCF4/LEF binding-sites for activating (α)-promoter, while activated NF-κBp65 (bound to NF-κB-*cis*-element), activates (β)-promoter in cancer-cells. DCLK1-S expression was examined in a cohort of 92 CRC patients; high-expressors had significantly worse overall-survival compared to low-expressors. Our novel findings’ regarding usage of alternate (β)-promoter by hCRCs, suggests that DCLK1-S may represent an important target for preventing/inhibiting colon-cancers, and for eliminating colon-CSCs.

CRC is the third most prevalent cancer in the U.S^1^,^2^. Several cancer stem cell (CSC) markers have been identified in literature, including CD44, CD133, Lgr5 and DCLK1^3^,^4^,^5^,^6^,^7^,^8^. Besides marking the cancer cells, CD44, CD133 and Lgr5 have been reported to play an important functional role in either maintaining the growth of the cancer cells and/or in aiding the metastatic potential of the cells^3^,^4^,^5^,^6^. More recently, an equally important role of DCLK1 has been implicated in colon tumorigenesis in mice^8^,^9^,^10^ and in maintaining the proliferative potential of human colon cancer cells^11^,^12^,^13^. We recently reported that a subset of DCLK1^+^ CSCs were resistant to inhibitory effects of chemopreventive/chemotherapeutic agents, and down-regulation of DCLK1 combined with chemoprevention was required for eliminating CSCs, *in vitro* and *in vivo*, and for avoiding relapse (in terms of re-formation of tumorospheres from treated cells)^11^. These findings highlighted a possible critical role of DCLK1 in maintaining the growth of human colon cancer cell lines. Isogenic clones of human embryonic epithelial cells (HEK293), that were either poorly tumorigenic (HEKC) or highly metastatic (HEKmGAS), expressed identical set of markers, including DCLK1^12^. Thus, specifically targeting CSCs has remained a challenge.

*DCLK1*-gene encodes a member of the protein kinase family and double-cortin family^14^, and was initially reported to play a critical role in neurogenesis and neuronal migration^14^,^15^,^16^. Thereafter, investigators reported an important role of DCLK1 in dictating cognitive behavior of mice and humans^16^,^17^. A possible important role of DCLK1 in maintaining tumorous growths was first learned from experiments with neuroblastomas^18^,^19^. Only in the past 7–8 years, epithelial expression of DCLK1 was described for the first time in mouse gastric epithelial cells^20^, and the authors speculated that DCLK1 was being expressed by gastric stem cells. Soon afterwards, laboratories of Drs. Anant and Houchen published several papers describing DCLK1 expression in mouse intestinal crypts^7^,^21^. Expression of DCLK1 in mouse colonic crypts was reported to be significantly elevated in response to progastrins (potent mitogens for colonic epithelial cells and colon cancers^22^,^23^, which correlated with hyperproliferation of the crypts^22^). DCLK1 is also expressed by acetylated Tuft cells, located in the upper 1/3 of colon crypts in mice^24^. More recently, a critical role of DCLK1^+^ Tuft cells was reported in developing colon and pancreatic tumors/lesions in mutant mouse models of carcinogenesis^9^,^10^. DCLK1^+^ Tuft cells were reported to be required for restitution of mouse intestinal crypts in response to inflammation/radiation damage^25^. Thus the literature so far strongly implicates a possible important role of DCLK1 in mouse colon tumorogenesis and in maintaining the growth of human colon cancers.

A number of long (~80–82 KDa) and short (~45–50 KDa) isoforms of DCLK1 have been identified in human brains/neurons^26^,^27^,^28^,^29^,^30^ (Supplementary Figs 1, 2). The ~82 kDa long isoform of DCLK1 contains: two N-terminal doublecortin domains which bind microtubules, a C-terminal serine/threonine kinase domain with homology to Ca^2+^/calmodulin dependent protein kinases and a middle serine/proline rich domain, which mediates protein interactions. The nomenclature for the various isoforms has remained a source of confusion, and differs even in the Swiss-Prot and NCBI databases (described in the legends of Supplementary Figs 1,2). The specific biological function of the various isoforms has remained undefined. The shorter isoforms lack the two N terminal doublecortin domains. Thus the 3D structure of the long vs short isoforms can be expected to be quite different, with perhaps some differences in their biological interactions and activities. The longer isoforms and their splice variants are presumed to be transcriptionally regulated by the 5′(α)-promoter. The origin of the shorter isoforms has not been investigated to a significant extent, but a 3′ promoter (termed β-promoter^28^), downstream of the 5′(α)-promoter has been implicated in transcribing shorter-transcripts of DCLK1 in mouse cerebellum^31^. In at least one report, a TATA box containing promoter was described in the IntronV of *DCLK1*-gene in neuronal cells^17^. Unlike the neuronal cells, possible expression of different isoforms of DCLK1 by normal colonic epithelial cells and colon cancer cells/tumors has not been investigated to-date. The presence of DCLK1 protein in epithelial cells has so far been mainly examined by using commercial antibodies, generated against the common C terminal end of long and short isoforms^11^,^12^,^32^,^33^,^34^. Thus the specific isoform(s) being expressed by epithelial cells has remained unknown.

In studies with mutant mouse models of colon/pancreatic tumorigenesis, described above, a *bac* construct, expressing either the reporter gene or diphtheria toxin, downstream of the 5′promoter of mouse *DCLK1* gene was used, suggesting that 5′promoter remains functional during intestinal/pancreatic tumorigenesis in mice, which likely results in the expression of the long isoform(s). The 5′promoter of h*DCLK1*-gene, however, was recently reported to be hypermethylated in hCRCs, by several investigators^35^,^36^, suggesting the possibility that the 5′promoter of h*DCLK1*-gene may be epigenetically silenced in hCRCs. This intriguing possibility was examined in the current studies, and our findings suggest that hypermethylation of 5′promoter is an early event during adenoma-carcinoma sequence of colon carcinogenesis in humans, unlike mice. Our data also suggests an absence of expression of long transcripts/isoforms in all 15 human colon cancer cell lines (hCCCs) screened to-date by us, suggesting epigenetic silencing of the 5′(α)-promoter due to its hypermethylation in hCRCs, as described above.

Even though the 5′(α)-promoter is epigenetically silenced in hCCCs/hCRCs, high levels of DCLK1 protein have been reported in hCCCs/hCRCs^11^,^37^,^38^,^39^. The discrepancy between the reported presence of DCLK1 protein in hCCCs/hCRCs, but hypermethylation/epigenetic silencing of 5′(α)-promoter, suggests the possibility that hCCCs/hCRCs may be utilizing an alternate promoter for expressing alternate isoforms of DCLK1. This novel possibility was examined as described below.

*In silico* analysis of h*DCLK1* gene, led us to confirm the presence of a canonical TATA box within the β promoter located within IntronV. We report for the first time, that IntronV-(β)promoter is used as an alternate-promoter by hCCCs/hCRCs for expressing a short transcript. Based on sequence homology, the long (L) and short (S) transcripts of DCLK1, found in normal human colon cell lines/normal human colons (hNCs) vs hCCCs/hCRCs, respectively, were determined to be identical to isoforms 1 (NM_004734.4) and 2 (NM_001195415.1) in the NCBI data base. For the purpose of our studies we have termed the isoform 1 as DCLK1-L and the isoform 2 as DCLK1-S, to clearly differentiate between the molecular size of the two isoforms. Colon tumors and normal colons from mice, on the other hand, were confirmed to only express the long isoform(s).

Transcriptional regulation of the α/β promoters in the h*DCLK1*-gene in epithelial cells remains largely unknown. Activation of β-catenin and NF-κBp65 was reported to be critically required for up-regulating DCLK1 protein in response to autocrine and endocrine progastrins^22^. We therefore conducted *in silico* analysis of the two promoters followed by promoter-reporter/ChIP assays, in the presence or absence of the known activator (progastrin), and report for the first time an important role of β-catenin binding to TCF4/LEF binding-sites for activating 5′(α)-promoter, and an important role of NF-κB binding-site for activating IntronV-(β)promoter.

In order to define pathophysiological relevance of DCLK1-S expression by hCRCs, the overall-survival of a cohort of 92 CRC patients was examined in relation to high/low expression of DCLK1-S. A clinically important finding was that high-expressors of DCLK1-S had significantly worse overall-survival, and disease free interval. DCLK1-S expression represented an independent diagnostic/prognostic marker for CRC patients.

## Results

### 5′-(α)promoter is methylated during colon-carcinogenesis in human

**Figure 1:**
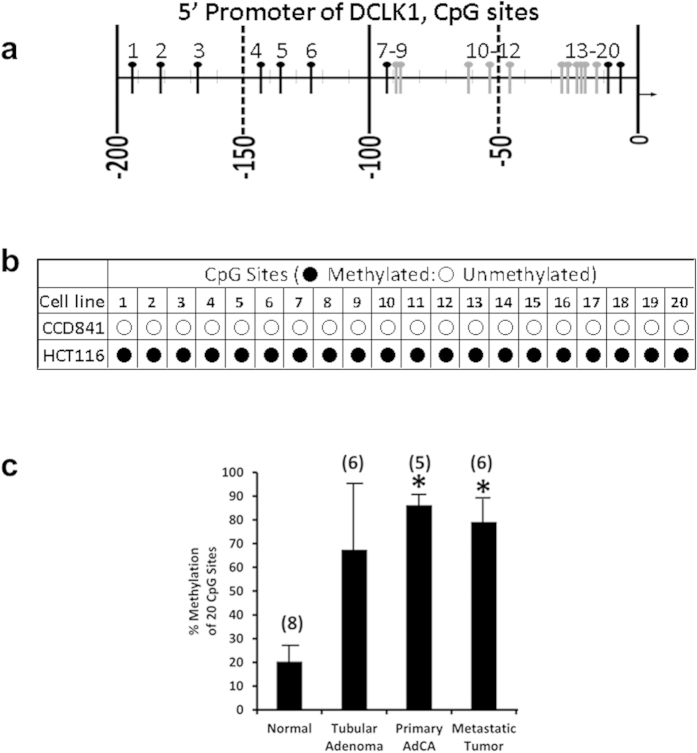
Methylation of 5′(α)-promoter of h*DCLK1*. (**a**) CpG sites that can be potentially methylated within 200bps of the 5′**(α)**-promoter of *DCLK1-gene* are depicted as vertical black/grey lines, and numbered 1–20. Grey vertical lines depict CpG sites used for assessing DNA methylation of 5′**(α)**-promoter of *DCLK1*-gene in a recent study^35^. (**b**)  Methylation status of 20 CpG sites was determined using the bisulfite method of sequencing as described in Methods. Methylation status of the 20 sites is shown for representative normal (CCD841) and colon cancer (HCT116) cell lines. Open circles = unmethylated CpG sites; filled circles = methylated CpG sites. (**c**) Methylation status is presented as bar graphs, and represents % CpG sites methylated (of the 20 sites analyzed) in samples from normal colons (Normal), colonic tubular-adenomas (TAs), primary adenocarcinomas (AdCAs), and metastatic (MET) tumors. Each bar graph = mean ± SEM of data from the indicated number of samples in parentheses, that were analyzed. *p < 0.05 vs methylation status of normal-colons that were obtained from patients free of adenomas and adenocarcinomas. The procurement of samples is described in methods.

In preliminary studies we discovered that 5′(α)-promoter of *DCLK1*-gene was hypermethylated in hCCCs, as recently reported^35^. We mapped a total of 20 CpG sites within 200 bps of the 5′(α)-promoter (Fig. 1a). All the 20CpG sites were non-methylated in the human normal colon (hNC) cell line (CCD841), but were methylated by >80% in 5 hCCC-lines, examined to-date. Mapping of the methylation status of individual CpG sites obtained from representative cell lines, by bisulfite sequencing is diagrammatically presented in Fig. 1b. Samples obtained from either normal (Norm) colons, adenomas (Ad), adenocarcinomas (AdCA) or metastatic-lesions (Met), from 5–8 patients, were also analyzed for methylation status of the indicated CpG sites, as described in Methods, and data from representative samples are presented diagrammatically in Supplementary Fig. 3. The percentage of 20 CpG sites, that were methylated in all the samples examined, was in the order of: AdCA/Met(85 ± 15) >TA(67 ± 30) >Norm (19 ± 8%) (Fig. 1c).

### Human normal colons (hNCs)/cells mainly express long-isoform of DCLK1 while hCCCs/hCRCs mainly express short-isoform

Hypermethylation of 5′-promoter of some genes during neoplastic-transformation is associated with expression of shorter transcripts from an alternate promoter^40^,^41^. Since 5′(α)-promoter of the *DCLK1*-gene is hypermethylated in hCRCs, but DCLK1 protein is measured in hCRCs, usage of an alternate-promoter was suggested.

**Figure 2:**
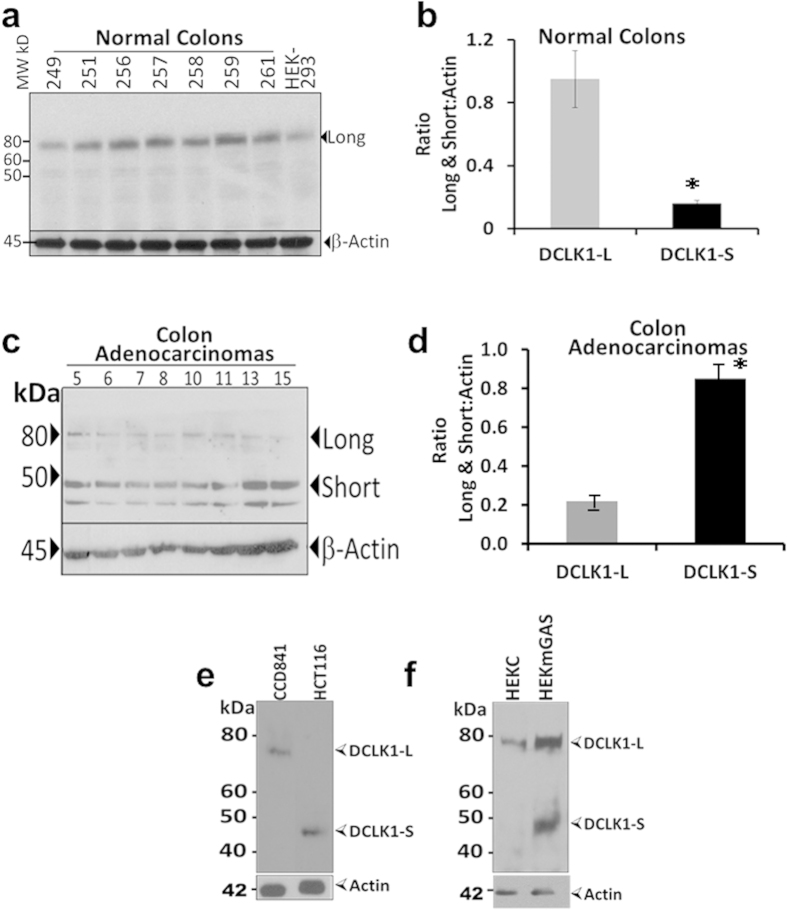
Western Blot (WB) analysis of DCLK1 protein in human cell lines and patient samples. The Mr of the proteins correspond to the long (isoform 1 in NCBI database) (~80 KDa) and short (Isoform 2 in NCBI database) (~45 KDa) DCLK1 protein in human cells. (**a–f**) Tissue samples were obtained from patients with either normal-colons (hNCs), free of Ads/AdCAs, or from patients with adenocarcinomas, as described in methods. Representative WB of samples from hNCs (**a**) and hCRCs (**c**) are presented, demonstrating relative expression of L/S DCLK1; laboratory numbers for patient samples are indicated above the Blots in (**a,c)**. WBs in each case were densitometrically analyzed and ratio of relative levels of L/S DCLK1 to corresponding β-actin levels are presented as bar-graphs, from all samples analyzed ((**b**) normal-colons; (**d**) adenocarcinomas). Each bar-graph in (**b**,**c**) mean ± SEM of calculated ratios for the two isoforms in patient samples obtained from 8–22 patients, as described in methods. (**e**) Representative Western Blots from normal (CCD841) and colon cancer (HCT116) cell lines. (**f**) Representative WBs from isogenic HEKC/HEKmGAS cells. Western blots presented in (**a**,**c**,**e**,**f**) were cropped to improve clarity. All bands within the range of the molecular markers were retained and processing of the film was applied equally across the entire image.

Molecular mass of DCLK1 was determined by Western Blot (WB) analysis using DCLK1-antibodies, which detect isoforms 1&2 in human brain. Almost all normal colonic mucosal samples (hNC) from patients were positive for the ~82 kDa DCLK1 protein, corresponding to long isoform (isoform 1 in NCBI data base) of hDCLK1; Less than 10% samples (1/22) were also strongly positive for S-isoform (Supplementary Table 2), which may be of prognostic value, since the patient was positive for large adenomas. Representative WB data from hNC patient samples are presented in Fig. 2a. A minor band of S-isoform was also seen in a few hNC samples (Fig. 2a,b; Supplementary Table 2), which may reflect expression of the short isoform by stromal and enteric neuronal cells, present within the colonic mucosa. The AdCA samples from patients with hCRCs were predominantly positive for ~45–48 kDa DCLK1 protein, corresponding to short(S) isoform (isoform 2 in NCBI data base) of hDCLK1. Representative WB data from AdCA samples in presented in Fig. 2c. The ratios of S/L DCLK1 to β-actin in hNCs vs hCRC samples, demonstrated opposite profiles (Fig. 2b,d). A hNC cell line (CCD841) only expressed DCLK1-L while HCT116 hCCC only expressed DCLK1-S (Fig. 2e). All 15 hCCC cell-lines, examined by RT-PCR, were negative for DCLK1-L; but the majority (13/15) expressed DCLK1-S (Supplementary Table 3). Representative RT-PCR data from hCCC cell-lines, wild type or mutant for KRAS/BRAF, are presented in Supplementary Fig. 4; the expression of DCLK1-S did not appear to be associated with any specific mutant phenotype of hCCC-cell lines. HEK293 cells, transduced to over-express progastrin (HEKmGAS), develop tumorigenic/metastatic potential^12^, and express elevated levels of both S/L DCLK1; control non-tumorogenic, HEKC cells, however, only express DCLK1-L (Fig. 2f). Thus, tumorigenic-transformation alone can apparently up-regulate the expression of the short-isoform, in the absence of epigenetic-silencing of 5′(α)-promoter.

**Figure 3:**
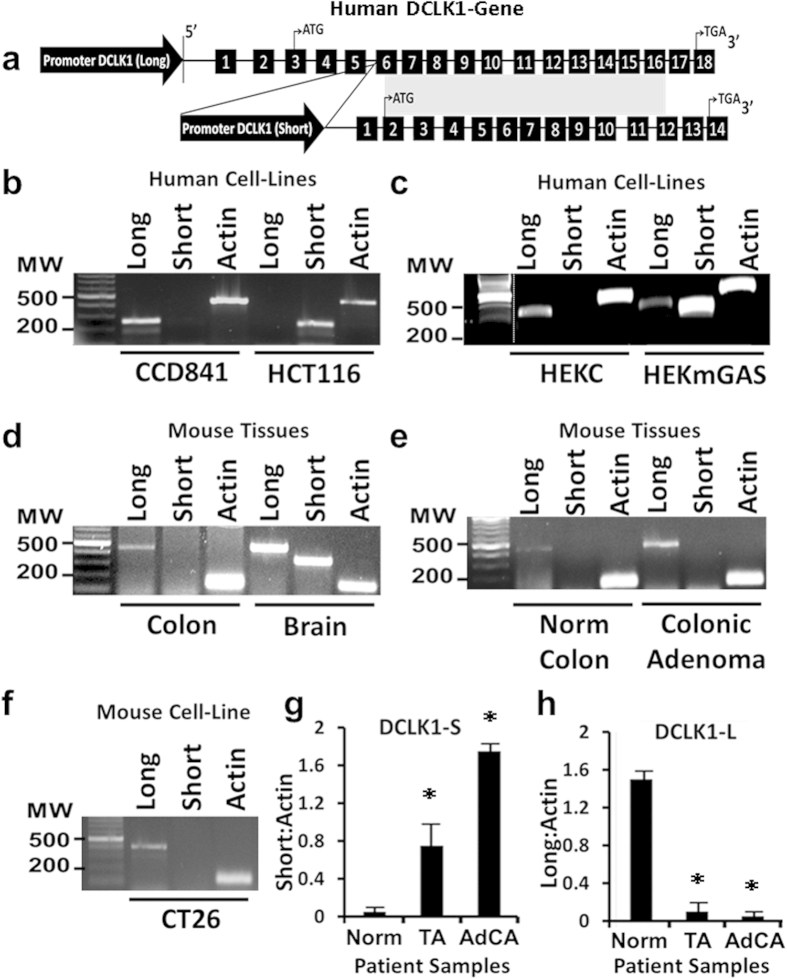
RT-PCR analysis of long and short transcripts of DCLK1 in human and mouse cell lines and in patient samples. (**a**) Diagrammatic representation of h*DCLK1* gene with transcription of DCLK1-L/S transcripts from the indicated exons (see Supplementary Fig. 1 for details). The α promoter for DCLK1-L is located at 5′-end and the alternate β promoter for DCLK1-S is located within IntronV of the gene. Transcriptional start sites (ATG) and end sites (TGA) are shown and homologous sequences between the two transcripts are shaded. Numbered boxes = exons; lines between boxes = introns. (**b–h**) Samples from mice and humans (patients) were obtained as described in methods and processed for RT-PCR using human/mouse primers for DCLK1-L/S transcripts. Representative RT-PCR data are shown from: human cell lines **(b,c)**; Normal-colonic-mucosa and brain tissues from wild type FVB/N mice (**d**); uninvolved mouse colon-mucosa (Norm) and mouse colon-tumor samples (Ads) (**e**); mouse cancer cell line (CT26) (**f**). Human (**b,c**) and mouse (**d–f**) β-actin was run as internal controls. The molecular weight (MW) in terms of bps is shown on left-hand side of each image in (**b–f**). Representative RT-PCR data from patient samples are presented in Supplementary Fig. 5a. Relative levels of short (**g**) and long (**h**) DCLK1 transcripts in human patient samples are presented as a ratio of the corresponding β-actin levels; hNC samples = Norm, tubular-adenomas = TA and colon-adenocarcinomas = AdCAs. Each bar-graph in (**g**,**h**) mean ± SEM of 5–8 separate patient samples, analyzed in duplicate. Electrophoresis gels presented in (**b–f**) were cropped to improve clarity. Processing of the electrophoresis blots was applied equally across the entire image. Touch-up tools were not used to manipulate data. Electrophoresis gels were cropped at the 100 bp and 1000 bp markers and all gels retain all experimental bands within the range of the molecular markers, co-run with the samples. In c, the dashed line represents additional cropping of the image where data, not included, was removed.

Genomic structure of h*DCLK1*-gene was mapped from contig NC_40000013.1 (Fig. 3a). Primer sets were designed for isoforms listed in NCBI database, to identify the isoforms being expressed by normal/non-transformed (CCD841/HEKC) and transformed (HCT116/HEKmGAS) cells. Long (NM_004734.4) and short (NM_001195415.1) transcripts, transcribed from the indicated exons (Fig. 3a), were detected (Fig. 3b,c). Only the 5′UTR and 17 bps, downstream of ATG, are non-homologous in S vs L transcripts; the rest of the coding sequence for *DCLK1-S* is homologous with *DCLK1-L* (Fig. 3a; Supplementary Fig. 1). Amino acid sequence of DCLK1-S was also >98% homologous with C-terminus of DCLK1-L (Supplementary Fig. 2a,b). We took advantage of slight differences in nucleotide sequences of L/S DCLK1, and developed isoform specific primers for amplifying L/S transcripts from human/mouse samples (Supplementary Table 1). HCT116 cells only expressed DCLK1-S, while normal CCD841 cells only expressed L-transcript (Fig. 3b). Non-tumorigenic HEKC cells only expressed L-transcript, while tumorigenic/metastatic HEKmGAS cells expressed both DCLK1-L/S (Fig. 3c), corresponding to protein data (Fig. 2f). Both L/S transcripts were expressed in mouse brain (Fig. 3d), as reported^27^, but mouse colonic epithelium only expressed Dclk1-L (Fig. 3d). Unlike hCRCs, 5′-promoter of m*Dclk1* gene does not appear to be epigenetically silenced in intestinal/pancreatic tumors^8^,^9^,^10^ as recently confirmed^42^. Norm/Ad samples from mouse colons (generated as described in methods), were subjected to RT-PCR, using mouse primers (Supplementary Table 1), and only L-transcript was amplified in both (Fig. 3e). In a mouse cancer cell line (CT26), only L-transcript was amplified (Fig. 3f). Thus, even though 5′-promoter of many common genes are epigenetically silenced in both mouse/human colon tumors^43^, 5′(α)-promoter of h*DCLK1* gene is silenced only in human colon tumors, as recently confirmed^35^. The loss or gain of DCLK1-L/S transcripts during different stages of colon-carcinogenesis was examined in patient samples, and representative RT-PCR data are presented in Supplementary Fig. 5a. Data from all samples (Fig. 3g,h), show that hNCs from patients mainly express L-transcript, while adenomas/adenocarcinomas mainly express S-transcript, albeit at significantly different levels. The fold-change in DCLK1-S expression by hCRC samples, at stages I-III, was examined by qRT-PCR, compared to that in hNCs, free of colonic growths (Supplementary Fig. 5b); higher levels were measured at stages I/II than stage III in the four samples analyzed/stage, using a commercial cDNA plate.

### Identification of transcriptional start site of DCLK1-transcripts in normal vs cancer cells

**Figure 4:**
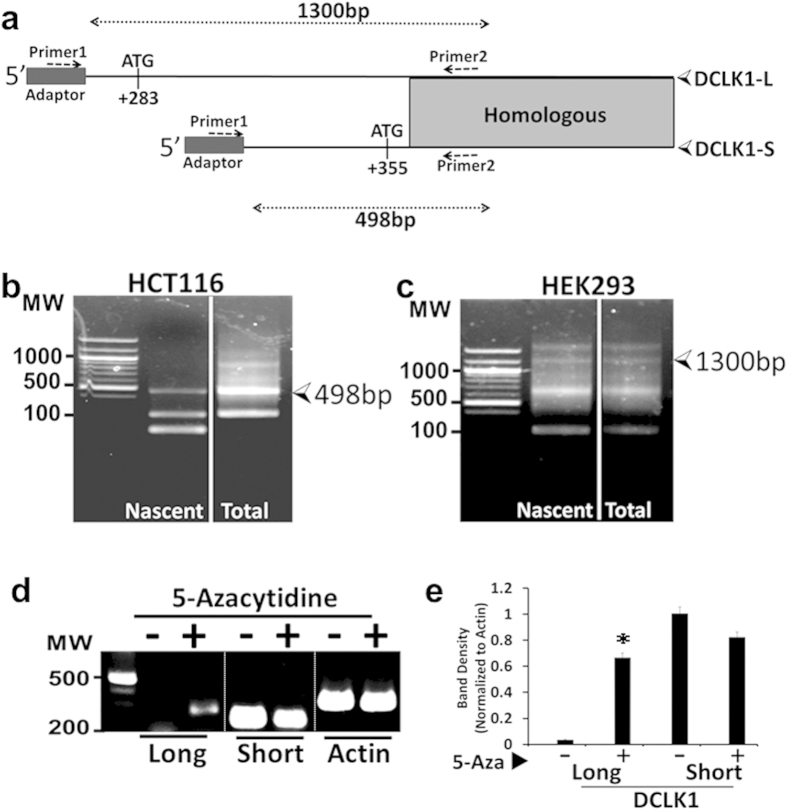
(**a–c**). Primer extension analysis for confirming transcription of DCLK1-L/S transcripts. **(a)** Schematic representation of 3′–5′ primer-extension analysis. The shaded portion shows 100% homology in the sequences between the two isoforms. A common primer-2 from the two transcripts was used for 3′–5′ extension, followed by ligating with non-mammalian adapter sequence (black box), as described in methods. Primers 1 and 2 were used for PCR amplification of the products. (**b,c**) Both nascent mRNA and total RNA were used for primer extension analysis, followed by PCR amplification, as described above. Resulting PCR products are presented in (**b**) (HCT116 cells) and (**c**) (HEK-293 cells). HCT116 cells were positive for only the short transcript (498bps) and HEK-293 cells were positive for only the long transcript (1,300bps). All other bands were either non-specific or fragments thereof, as confirmed by sequencing. Electrophoresis gels shown in b and c, were not cropped. A white line is provided to clearly separate the nascent RNA from the total RNA. (**d–e**) Confirmation of epigenetic silencing of 5′(α)-promoter ofDCLK1-gene in HCT116 cells. Relative expression (RT-PCR) of L/S DCLK1 in HCT116 cells, in presence or absence of treatment with 5‐aza‐2′‐deoxycytidine (5-azacytidine) is shown. (**d**) Representative RT-PCR data. Image was cropped to present data within the range of the molecular markers as described in the legend of Fig. 3. (**e**) densitometric data presented as % of β-actin in corresponding samples. Each bar-graph in (**e**) mean ± SEM of three experiments.

A common reverse-primer (primer-2) from coding sequence of L/S transcripts was designed (Supplementary Table 1), and either nascent-mRNA or total-RNA was reverse transcribed, as diagrammatically shown (Fig. 4a). A non-mammalian adapter-sequence was ligated to the products and PCR amplified using primers 1/2 (Fig. 4a); results are shown in Fig. 4b,c. HCT116 cells only expressed a 498 bp-product, matching the expected size of short-isoform (NM_001195415.1) (Fig. 4b). HEK293 samples only expressed a 1,300 bp-product, matching the expected size of DCLK1-L transcript (NM_004734.4) (Fig. 4c). Sequencing confirmed the expected products. All other bands were fragments thereof or non-specific. The results confirm that hCCCs express DCLK1-S from the β promoter in IntronV of h*DCLK1*-gene. HCT116 cells, treated with 5-Azacytidine, re-expressed DCLK1-L transcript (Fig. 4d,e), confirming that 5′-promoter of h*DCLK1* gene is epigenetically-silenced in HCT116 cells.

### Role of TCF4/LEF binding-sites in up-regulating transcriptional activity of 5′(α)-promoter of hDCLK1 gene

**Figure 5:**
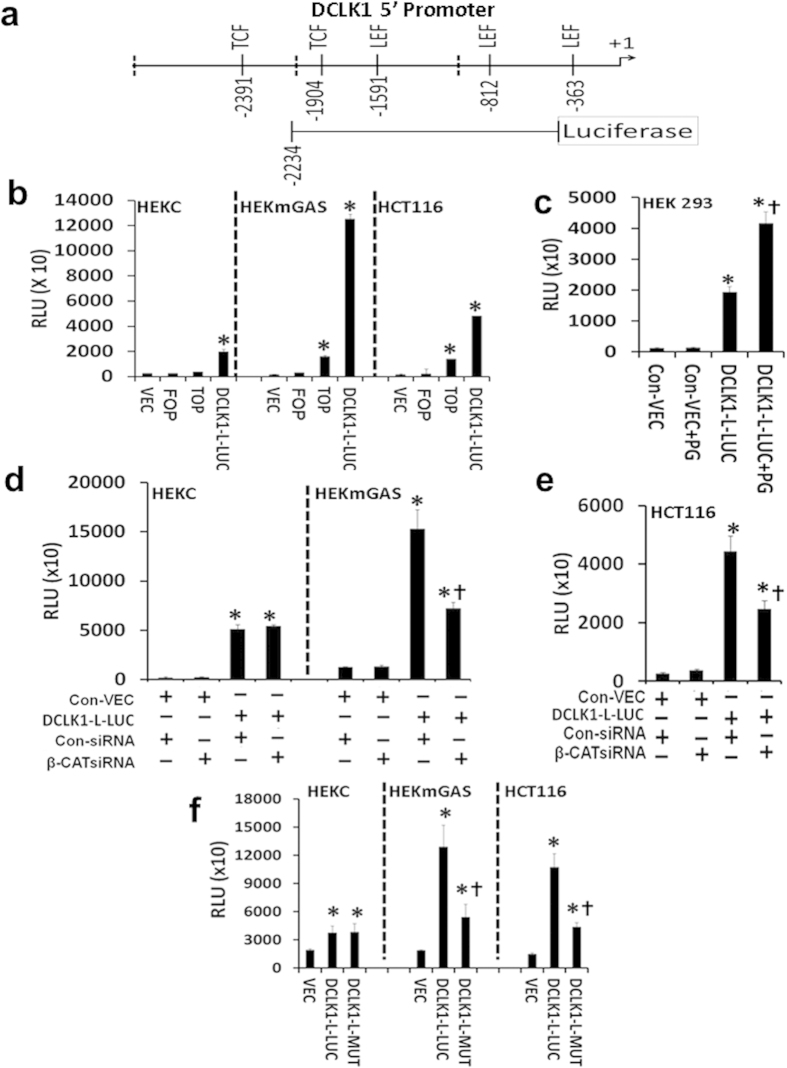
Role of TCF4/LEF binding-sites in activation of 5′(α)-promoter. (**a**)  *In silico* analysis of ~3 kb of 5′**(α)**-promoter (transcribing DCLK1-L), identified several binding sites for TCF-4/LEF and NF-κB, with > 90% conserved sequences. The construct (*DCLK1*-L-LUC) used for the promoter-reporter assays is diagrammatically shown below the mapped promoter. **(b–f)**  Relative transcriptional/luciferase activity (RLU) in the indicated cells, transiently-transfected with the plasmids for 48 h, in the presence or absence of transfection with either PG expressing plasmid (p-mGAS) **(c)**, or the indicated siRNA (**d,e**), or a mutant plasmid (DCLK1-L-Mutant), containing insertions in the −1904/−1591 TCF/LEF binding sites (**f**), as shown. Methods used to generate the mutant plasmid are described under methods section. Cells were co-transfected with promoter-reporter construct ± p-mGAS/siRNA for data presented in (**c–e**). VEC = control LUC vector; TOP = TOPFlash plasmid with wild type TCF4/LEF binding sites for β-catenin binding; FOP = mutant FOPFlash plasmid. Each bar-graph in (**b–f**) represents mean ± SEM of three separate experiments conducted in duplicate or triplicate/experiment. *p < 0.05 vs corresponding values with control vector. † in (**b**) p < 0.05 vs corresponding values in HEKC cells; † in (**c**) p < 0.05 vs corresponding values with *DCLK1*-L-LUC vector alone. † in (**d**,**e**) p < 0.05 vs control siRNA values; † in (**f**) p < 0,05 vs wild type DCLK1-L-LUC values.

We used progastrin (PG) as an activator of DCLK1 expression in target cells, based on previous findings^22^,^23^. PG is a potent co-carcinogen and increases colon-carcinogenesis in mice, in response to AOM ± DSS^23^,^44^,^45^. Two potent transcription-factors (TFs) (NF-κBp65/β-catenin) mediate hyperproliferative/co-carcinogenic effects of PG on mouse colonic crypts^22^,^46^,^47^, associated with significant up-regulation of stem-cell-markers, including DCLK1^22^,^23^. Since colon-carcinogenesis in mice is associated with increased expression of Dclk1-L (Fig. 3), and NF-κB/β-catenin mediate up-regulatory effects of PG^22^,^46^,^47^,^48^, we conducted *in silico* analysis of 5′**(α)**-promoter. Several potential binding-sites for TCF4/LEF, and NF-κB, were found within 5 kb of start-site (Fig. 5a, Supplementary Fig. 6a). A 5′-promoter-reporter construct, containing TCF4/LEF and NF-κB binding-sites, was generated. Relative transcriptional-activity of promoter-reporter construct was examined in transiently transfected HEKC/HEKmGAS/HCT116 cells (Fig. 5b). CCD841 cells were not used as they were difficult to transfect. Corresponding levels of activated β-catenin were indirectly examined by measuring relative activation of TOP vs FOP plasmids, as described in Methods. Non-tumorigenic HEKC cells demonstrated relatively low levels of activated β-catenin (TOP-activity), while HEKmGAS/HCT116 cells were positive for significant levels of activated β-catenin/NF-κB (Fig. 5b, Supplementary Fig. 6b), probably in response to autocrine PG^22^. Transcriptional activity of 5′**(α)**-promoter-reporter construct (DCLK1-L-LUC) was several-fold higher in HEKmGAS/HCT116 cells compared to that in HEKC cells, suggesting that β-catenin binding to 5′**(α)**-promoter may contribute to increased activation of DCLK1-L-LUC vector (Fig. 5b). HEK293 cells were transiently co-transfected with either control-vector or mGAS-vector to express high levels of PG^12^,^22^, along with DCLK1-L-LUC vector. Transcriptional activity of DCLK1-L-LUC in the presence of PG expression was significantly increased in HEK293 cells (Fig. 5c). Transcriptional activity of DCLK1-L-LUC-vector was significantly reduced in HEKmGAS/HCT116 cells to control HEKC levels, on co-transfection with β-catenin siRNA (Fig. 5d,e); efficacy of β-catenin-siRNA was confirmed (Supplementary Fig. 7). Possible role of NF-κB-binding-sites in regulating 5′**(α)**-promoter was examined by co-transfecting HEKC/HEKmGAS cells with NF-κBp65-siRNA and DCLK1-L-LUC vector. Relative activity of DCLK1-L-LUC vector was similar in control-siRNA vs NF-κBp65-siRNA treated cells, corresponding to relative levels of DCLK1-L transcript in control vs treated cells (Supplementary Fig. 7b); the latter results strongly suggest that NF-κB-binding-sites do not play a significant role in activating/regulating the 5′**(α)**-promoter in these cells.

**Figure 6:**
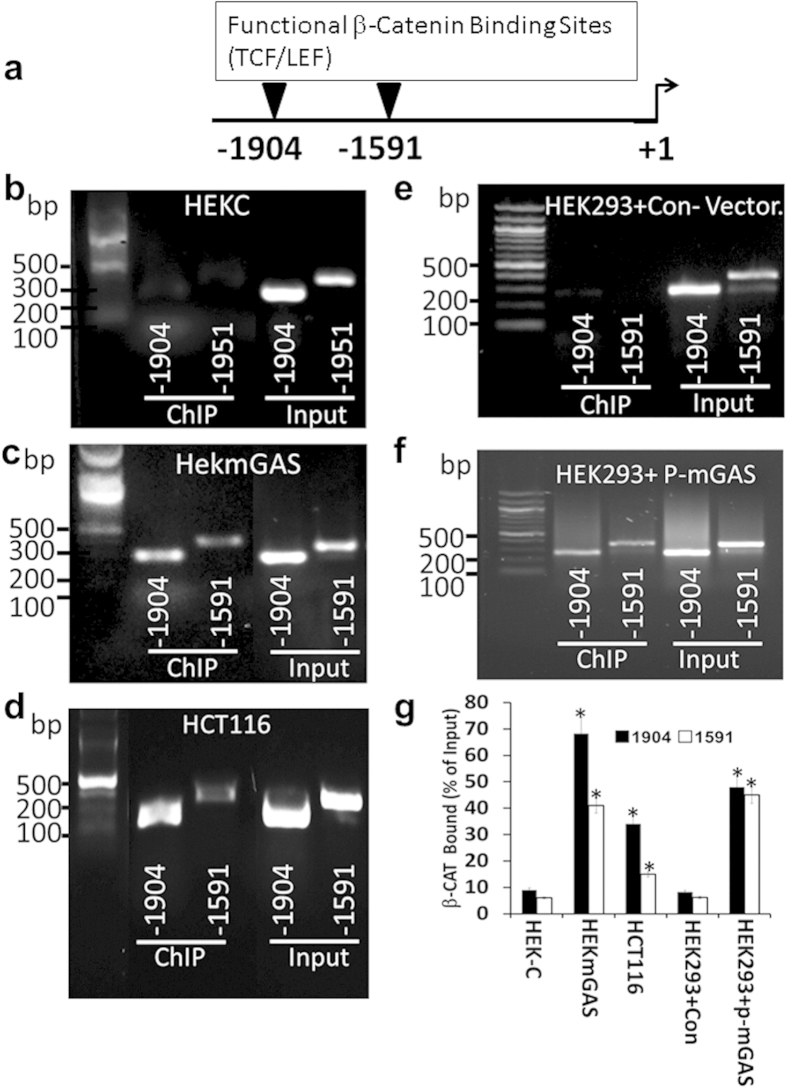
*In situ* binding of endogenous β-catenin to the two functional TCF4/LEF binding sites in the 5′(α)-promoter of *DCLK1*-gene. (**a**) Map of functional TCF4/LEF binding sites in 5′(α)-promoter of *DCLK1*-gene, as determined by ChIP analysis for β-catenin binding. (**b–d)**  Relative binding of β-catenin to the indicated TCF4/LEF binding sites in the indicated cell lines, by ChIP analysis. Total level of β-catenin in the samples is presented as input. Electrophoresis gels were not cropped. (**e,f**) Relative binding of β-catenin to functional TCF4/LEF binding sites in HEK-293 cells, transfected with either control vector (**e**) or PG expressing (p-mGAS) vectors (**f**), 48 h before ChIP. Data presented in b-f is representative of six observations from three experiments. (**g**) Relative binding of β-catenin, *in situ*, to functional binding sites in 5′(α)-promoter of DCLK1-gene, in different cell-lines, in presence or absence of mPG expression (described above), presented as % of input. Each bar-graph = mean ± SEM of duplicates from three experiments. % binding of β-catenin was determined by densitometric analysis of indicated bands.

β-catenin binding to the five potential TCF4/LEF binding-sites in 5′**(α)**-promoter (Fig. 5a), was determined in ChIP assays. TCF4/LEF sites at −1904 and −1591 were the only functional β-catenin binding-sites in the indicated cells (Fig. 6a). Representative ChIP data from all three cell-lines confirmed that non-tumorigenic HEKC cells, lacking activated β-catenin (Fig. 5b), were negative for β-catenin binding to both sites, while tumorigenic cell lines (HEKmGAS, HCT116) were positive (Fig. 6b–d). HEK293 cells were transiently transfected with either control or mGAS (PG expressing) vector, and analyzed by ChIP assays (Fig. 6e,f). Relative binding of β-catenin to the two TCF4 binding-sites, in the presence or absence of mGAS expression, from several experiments, is presented as % of total β-catenin (input) in the cells (Fig. 6g). β-catenin binding to both sites increased significantly in HEK293 cells transfected with mGAS-vector. For reasons unknown, relative binding of β-catenin to −1904 site was significantly higher than that to −1591 site in HEKmGAS/HCT116 cells (Fig. 6g).

To confirm a role of the −1904 and the −1591 TCF4/LEF binding-sites in transcriptional regulation of DCLK1-L-LUC vector, the two sites were mutated as described in Methods, and confirmed. All three cell lines were transfected with either the mutant DCLK1-L-Luc construct (termed DCLK1-L-Mutant) or the wildtype DCLK1-L-Luc construct. The transcriptional activity of DCLK1-L-Mutant construct was significantly down-regulated in HEKmGAS/HCT116 cells, to control levels measured in HEKC cells (Fig. 5f), mimicking the results obtained with the wildtype DCLK1-L-Luc construct in the presence of β-catenin siRNA (Fig. 5d,e). Results in Fig. 5f provide further evidence that the two TCF4/LEF binding sites play a critical role in transcriptional regulation of the 5′promoter.

### Role of NF-κB binding-site in regulating transcriptional activity of IntronV(β)-promoter of h*DCLK1*-gene

**Figure 7:**
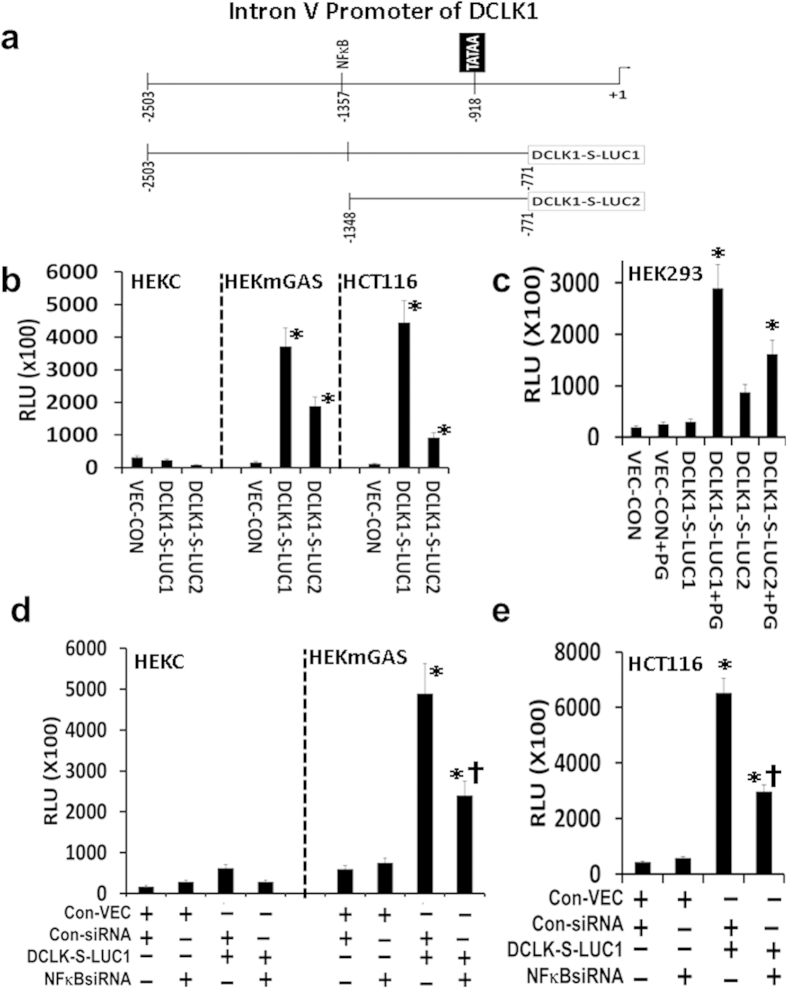
Role of NF-κB binding site in activation of IntronV(β)-promoter of *DCLK1* gene. (**a**)  *In silico* analysis of IntronV**(β)**-promoter demonstrated presence of a consensus TATA box and a consensus NF-κB binding site, as shown. IntronV**(β)**-promoter-luciferase constructs, used in the current studies, are diagrammatically shown as *DCLK1*-S-LUC-1 and *DCLK1*-S-LUC-2. (**b–e**) Transcriptional activity of promoter-reporter constructs in the indicated cell lines (**b**), in the presence or absence of PG expression (**c**) or treatment with either control or NF-κBp65-siRNA (**d,e**), as described in the legend of Fig. 5. Transcriptional activation in terms of luminescence (RLU) is presented in (**b–e**). Each bar-graph = mean ± SEM of data from three experiments, conducted in triplicate. *p < 0.05 vs control vector in (**b–e**). † in **(c)**  p < 0.05 vs LUC-1 or LUC-2 vector alone, in the absence of PG expression. † in (**d,e**) p < 0.05 vs corresponding data with control siRNA treated samples.

By *in silico* analysis, a single NF-κB binding site (~439 bps, 5′ of a consensus TATA box), but no TCF4/LEF sites, were identified within 3 kb of IntronV(β)-promoter (Fig. 7a). Role of NF-κB in regulating transcriptional activity of IntronV(β)-promoter was examined by using two promoter-reporter constructs (Fig. 7a). NF-κB cis-element was present in DCLK1-S-LUC-1, but absent in DCLK1-S-LUC-2 (Fig. 7a). Transcriptional activity of both promoter-reporter constructs was negligible in HEKC cells (Fig. 7b), known to be negative for activated NF-κBp65^22^. Relative transcriptional activity of LUC-1 was ~2–4 fold higher in HEKmGAS/HCT116 cells, compared to that of LUC-2 construct (Fig. 7b), suggesting an important role of NF-κB binding-site in mediating increased activation of IntronV(β)-promoter. Transcriptional activity of LUC-2 was also elevated in HEKmGAS/HCT116 cells (Fig. 7b), suggesting endogenous factor(s), other than p65, may also activate IntronV(β)-promoter. PG is overexpressed in hCRCs^38^,^49^, and maybe a prognostic marker for hCRC patients^50^. In the presence of PG (mGAS-vector), transcriptional activity of LUC-1 increased by ~10–15-fold in HEK293 cells (Fig. 7c), confirming an important role of NF-κB binding site in transcriptional activation of IntronV(β)-promoter in response to PG. Surprisingly transcriptional activity of LUC-2 (negative for NF-κB binding-site) was also increased by ~3–5-fold, suggesting that *cis*-elements other than NF-κB, might also respond to PG. Cells transfected with LUC1-vector were also co-transfected with either control- or NF-κBp65-siRNA (Fig. 7d,e). Loss of NF-κBp65 expression in NF-κBp65-siRNA transfected cells (Supplementary Fig. 7b), resulted in reduction of transcriptional activity of LUC-1 in HEKmGAS/HCT116 cells by >50% (Fig. 7d,e), to levels measured with LUC-2 (Fig. 7b). The results suggest that the single NF-κB *cis*-element plays an important role in transcriptional activation of IntronV**(β)**-promoter, and hence the expression of S-isoform, in transformed/cancer cells.

**Figure 8:**
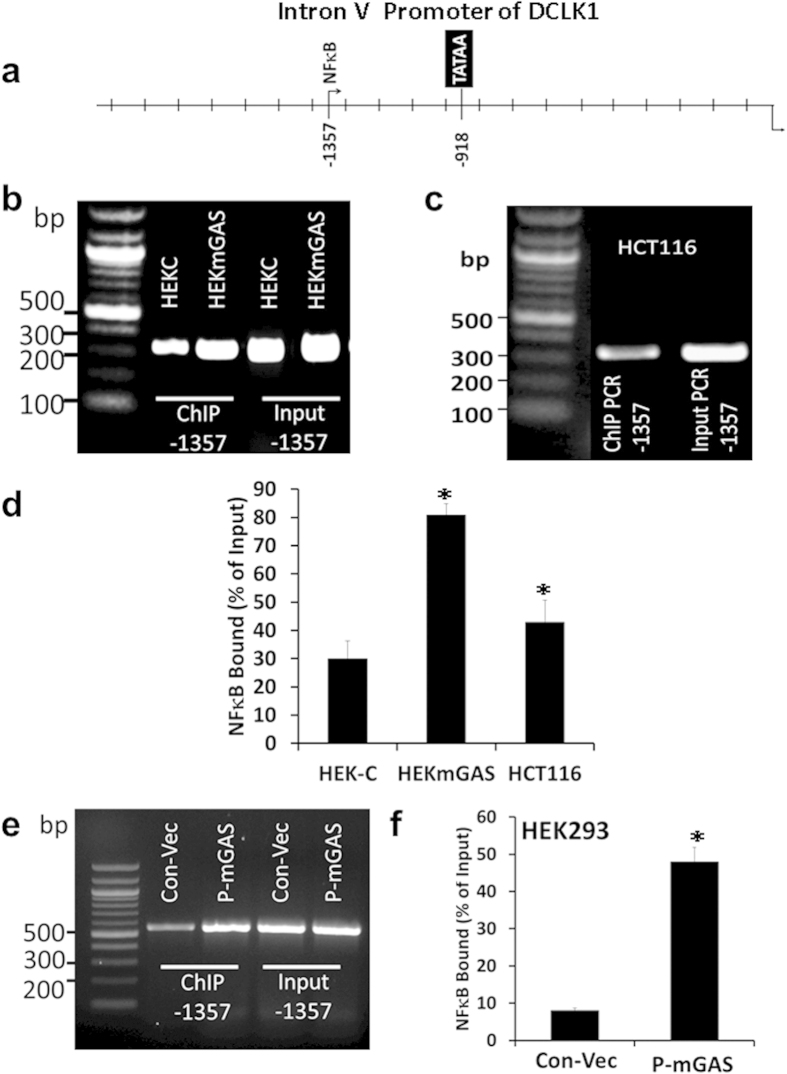
Binding of endogenous activated NF-κBp65 to the single NF-κB binding site in the IntronV(β)-promoter, *in situ*, in human cell lines. (**a**)  *In silico* analysis of IntronV(β)-promoter DNA, mapped a conserved NF-κB binding site within 500 bps of TATA box. (**b,c**) ChIP analysis, demonstrating relative binding of NF-κBp65 to the single NF-κB binding site in the indicated cell lines. (**d**) Relative levels of NF-κBp65 bound to NF-κB binding site was calculated as % of input by densitometric analysis of the bands from all experiments and are presented as bar-graphs for the indicated cell lines. (**e**) Relative binding of NF-κBp65 to the single NF-κB binding site, in the presence or absence of PG expression (p-mGAS), was measured by ChIP analysis, as described in the legend of Fig. 6. Electrophoresis gels were not cropped. (**f**) Relative levels of NF-κBp65 bound to the NF-κB binding site in HEK-293 cells, in the presence of PG expression, is presented as % of input, by densitometric analysis of the bands. (**b,c,e**) representative ChIP data from one of three experiments. Each bar-graph in (**d,f**) mean ± SEM of data from three experiments, run in duplicate.

Representative ChIP data confirms binding of NF-κBp65 to NF-κB binding-site in IntronV-promoter (Fig. 8a), *in situ* (Fig. 8b,c). Almost 80–90% of total NF-κBp65 was bound to NF-κB binding-site in HEKmGAS/HCT116 cells. Surprisingly, ~30–40% of total NF-κBp65 was also bound in HEKC cells (Fig. 8d), even though transcriptional activity of the promoter-reporter construct was negligible in these cells (Fig. 7b,d), suggesting that either a threshold of NF-κB binding is required, or other factors activate IntronV(β)-promoter, in the presence of NF-κBp65. The % bound NF-κBp65 increased by ~5-fold in HEK293 cells overexpressing PG (mGAS vector) (Fig. 8e,f), corroborating our previous findings of significant increase in phosphorylation/activation of NF-κBp65 in response to PG^22^,^48^.

### High expression of DCLK1-S in AdCA samples from CRC patients is associated with poor patient survival

**Figure 9:**
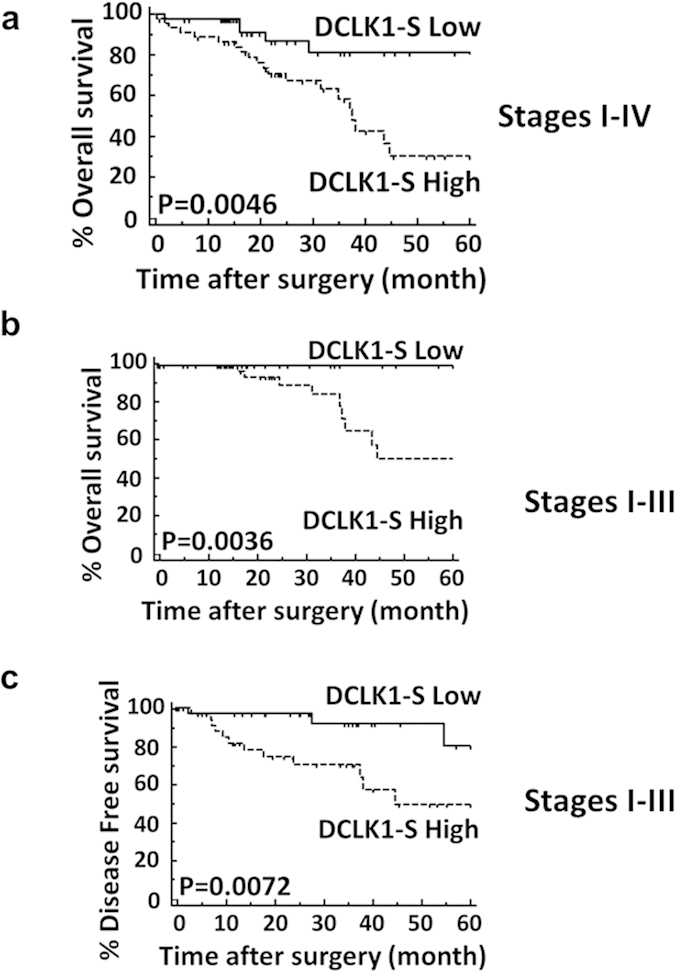
Overall survival and disease free survival of patients with CRC, in relation to low or high expression of DCLK1-S. (a) Kaplan-Meier overall survival curves of CRC patients, with stages I-IV disease in relation to relative expression levels of DCLK1-S measured by qRT-PCR. n = 92 patients. (**b**) Kaplan-Meier overall survival curves of CRC patients with stages I-III disease, in relation to relative expression levels of DCLK1-S measured by qRT-PCR n = 71 patients. (**c**) Kaplan-Meier disease free survival curves of CRC patients with stages I-III disease, in relation to relative expression levels of DCLK1-S measured by qRT-PCR. n = 67 patients. The cutoff threshold values were defined by using the median values of DCLK1-S expression of each cohort in cancer tissues.

The expression pattern of DCLK1-S transcript in relation to clinicopathological parameters was analyzed using an independent cohort of patient specimens, as described in Methods. High-expression of DCLK1-S significantly correlated with overall poor patient survival in patients with Stages I-IV disease (Fig. 9a), or patients with only curatively resected Stages I-III disease (Fig. 9b), with significantly worse disease free survival (Fig. 9c), which significantly correlated with pathological T-category and lymphatic vessel involvement (Supplementary Table 4). Moreover, by multivariate analysis, overexpression of DCLK1-S emerged as an independent prognostic factor in CRC patients (Supplementary Table 5).

## Discussion

A clinically important discovery of the current studies is that an alternate-promoter (β) within IntronV of *DCLK1* gene is used by human colon cancer cell lines (hCCCs) and hCRCs to express a short-transcript of DCLK1 (DCLK1-S) (termed Isoform 2 in NCBI data base). In a cohort of 92 patients, we found that high-expressers of DCLK1-S had an overall worse survival and disease free survival than low-expressers (Fig. 9). DCLK1-S expression was determined to be an independent prognostic factor for patients with CRCs (Supplementary Table 5). Another important finding was that epigenetic silencing of 5′(α)-promoter and loss of expression of DCLK1-L during adenoma-carcinoma sequence of colon-carcinogenesis was chronologically followed by activation of IntronV(β)-promoter of h*DCLK1*-gene, even though the two events are probably independent and not connected mechanistically.

We did not observe DNA-methylation of 5′(α)-promoter in HEKmGAS cells, suggesting that epigenetic silencing of 5′(α)-promoter is not a pre-requisite for activating IntronV(β)-promoter. Sustained activation of NF-κB, downstream of autocrine PG, may play an important role as well, as suggested by data in Fig. 7. Overexpression of PG in normal intestinal epithelial cells was ineffective towards imparting tumorigenic potential to the cells^45^, suggesting that overexpression of PG and activation of NF-κB pathway, in the context of human embryonic cells, up-regulates tumorigenic pathway which appears to include activation of IntronV(β)-promoter of h*DCLK1*-gene. Inflammatory microenvironment of tumors, potentially leading to sustained activation of NF-κB pathway, may also play a role in elevated levels of DCLK1-S in Ads/AdCAs, *in situ, (*Figs 2 and 3), as suggested in literature^51^. Thus, factors up-stream of activation of DCLK1-S expression, such as an inflammatory-microenvironment/progastrins/activation of oncogenic-pathways, likely play an important role in the expression of DCLK1-S in hCRCs.

A critical role of DCLK1 expression in maintaining tumorigenic/metastatic potential of hCCCs/CSCs was previously reported^11^,^13^. In the current studies, DCLK1-S was identified as the major isoform in hCCCs/hCRCs, with a few exceptions (Figs 2 and 3), suggesting that DCLK1-S likely supports the previously reported tumorigenic/metastatic potential of hCCCs^11^,^12^. However, in mouse models of colon-carcinogenesis, high levels of Dclk1-L in the absence of Dclk1-S are expressed (Fig. 3e). Co-expression of diphtheria-toxin in Dclk1^+^ cells in small-intestines/colons, results in loss of tumorigenesis in mouse models of colon carcinogenesis^8^,^9^. These findings suggest that Dclk1-L expression is required for colon tumorigenesis in mice. Metastatic spread of mouse colon tumors, however, has not been reported in Apc^Min/+^ mice or in mice treated with AOM ± DSS^8^,^9^,^44^,^45^. Epithelial-mesenchymal-transition by hCCCs requires DCLK1 expression^52^, suggesting that metastatic spread of colon cancer cells may require the expression of DCLK1-S by hCCCs, which only express DCLK1-S (Supplementary Table 3). We recently reported expression of DCLK1-S by circulating cancer-stem-cells in hCRC patients^53^, providing further evidence that DCLK1-S may be required for imparting metastatic potential to hCCCs. The latter possibility is further supported by the fact that, HEKmGAS cells overexpressing DCLK1-S (Figs 2 and 3), implanted in the cecum of athymic nude mice, metastasized to the liver^12^. Thus, metastasis of colon tumors is possible in mice, but absence of Dclk1-S expression by mouse tumors may impede metastasis. This intriguing possibility needs to be examined in future.

As discussed in introduction, DNA methylation and epigenetic-silencing of 5′(α)-promoters has been documented for many genes during tumorigenesis. Multiple promoters are methylated in both mouse tumors and hCRCs^43^. However, in a recent report^42^, it was confirmed that 5′(α)-promoter of some genes (including *DCLK1*) are methylated and silenced in human colon tumors, but not in mouse colon tumors. Reports in literature (as discussed in introduction) confirm that 5′(α)-promoter of mouse *Dclk1*-gene does not get silenced during tumorigenesis, as confirmed by us (Fig. 3). In the current studies, we further confirm that loss of DCLK1-L in hCCCs is due to DNA methylation and can be reversed with de-methylating agents (Fig. 4d,e). Normal human colon cell line and hNCs, on the other hand, continue to express DCLK1-L from 5′(α)-promoter. This important difference in hNCs and hCCCs was confirmed by primer-extension analysis (Fig. 4a–c). Majority of the hCCCs/CRCs up-regulate expression of DCLK1-S from an alternate(β)-promoter within IntronV, while mouse colon tumors do not (Fig. 3), for unknown reasons.

The activation of (β)-promoter for transcribing Dclk1-S isoform was recently described in mouse cerebellum^31^. The use of alternate-promoters for transcribing shorter isoforms, especially for genes which have hypermethylated 5′-promoters, is a dominant phenomenon and more common than transcription of splice-variants during development and disease progression^31^,^40^,^41^. There is thus accumulating evidence in recent literature which strongly supports our novel findings regarding the use of an alternate-(β)promoter within IntronV for expressing shorter isoforms of DCLK1 in hCCCs/hCRCs. More recently, shorter isoforms of DCLK1 (47KDa) were reported in KRAS mutant hCCCs^54^, which further supports our findings; however, we did not observe a specific correlation between expression of DCLK1-S and mutant phenotype of hCCCs (Supplementary Table 3).

By *in silico* analysis, we discovered that while the 5′(α)-promoter was positive for functional TCF4/LEF binding sites and a few NF-κB binding sites (Figs 5 and 6, Supplementary Fig. 6), the IntronV(β)-promoter was positive for a functional NF-κB binding site, upstream of a TATA box (Figs 7 and 8). We therefore examined the role of NF-κB/β-catenin signaling pathways in regulating the activity of α/β promoters. Since progastrins activates NF-κB/β-catenin signaling pathways^22^,^46^,^47^,^48^, resulting in increased expression of stem cell markers, including DCLK1 in normal colon crypts and transformed cells^12^,^22^, we used progastrin for activating NF-κB/β-catenin in HEK293/HEKC cells, and examined their role in activating 5′(α)-promoter for DCLK1-L expression. Since tumorigenic/metastatic potential of HCT116/HEKmGAS cells is dependent on autocrine PG^12^,^54^, we used these cell lines to examine the role of NF-κBp65 in mediating transcriptional activation of intronV(β)-promoter for expressing DCLK1-S. Experiments with promoter-reporter constructs along with ChIP assays, in the presence or absence of siRNAs against the two transcriptional factors (Figs 5, 6, 7, 8), confirmed that TCF4/LEF binding sites, in response to activated β-catenin, activates 5′(α)-promoter of Dclk1-L (in tissues such as mouse colons/tumors^22^,^23^), while NF-κB binding site, in response to activated NF-κBp65 and its partners, activates IntronV(β)-promoter (thus up-regulating DCLK1-S expression in hCCCs, Figs 2 and 3, Supplementary Table 3). NF-κB binding sites in the 5′(α)-promoter, on the other hand, did not appear to be playing any role in activating the (α)-promoter and/or the expression of DCLK1-L (Supplementary Fig. 6). Both the 5′(α) and IntronV(β) promoters are positive for several other binding sites, which likely play synergistic/antagonistic roles in dictating transcriptional activity of the promoters, which was not examined in the current study.

In summary, our findings suggest that the 5′(α)-promoter of *DCLK1*-gene becomes epigenetically silenced during colon-carcinogenesis at early stages, resulting in loss of expression of DCLK1-L in adenomas and hCRCs. Oncogenic and inflammatory pathways associated with colon-carcinogenesis may be involved in transcriptional-activation of the alternate-(β)promoter within IntronV, resulting in alternate expression of DCLK1-S. Usage of two separate promoters by normal vs cancer cells in humans, provides an opportunity for developing methods for specifically targeting DCLK1-S as an approach for eliminating colon cancer growths. Additionally, since high expressers of DCLK1-S had worse overall/disease free survival, DCLK1-S expression by colonic tumors may provide a useful diagnostic/prognostic tool.

## Materials and Methods

### Reagents used

Antibodies used in these studies included: anti-total-p65NF-κB, anti-β-catenin (total) (Cell Signaling Technology, Danvers, MA); anti-β-actin (total) (Sigma, St. Louis, MO); anti-DCLK1 antibody (Abcam AB31704, Cambridge, MA). Mono-specific rabbit polyclonal anti-progastrin-antibody and eukaryotic plasmid, expressing triple mutant human gastrin gene, for overexpressing human progastrin (PG) peptide, were generated in our laboratory as previously described^22^. Smart Pool of target-specific small interfering RNA (siRNA) and non-targeting (control) siRNA Pool were purchased from Dharmacon (Lafayette, CO). Sepharose beads and all other chemical reagents were purchased from Sigma. TissueScan^TM^ Disease Tissue qPCR array (Catalogue Number HCRT102) for colon cancer and normal colons was purchased from OriGene (Rockville, MD). cDNA synthesis master mix was purchased from GeneDEPOT (Baker, TX). Syber green qRT-PCR kit was purchased from Bio-Rad (Hercule, CA). Promega GoTaq®green Master Mix (Maddison, WI) was used for PCR amplification, using a Thermal Cycler from Eppendorf (Hauppauge, NY). Cloning vector pGL2 was from Promega, and TOPO-TA cloning vector was purchased from Invitrogen (Grand Island, NY). Restriction enzymes and competent cells were purchased from New England BioLabs (Ipswich, MA). Transfection reagent FuGENE®6 was bought from Roche (Branford, CT), and all primers used were synthesized by Sigma.

### Cell culture

HEK293 and HCT116 cell lines were obtained from ATCC, and have been maintained in the laboratory for several years. CCD841 and CT26 cells were generously gifted to our laboratory from Dr. Carla Kantara (Department of BMB, UTMB) and Dr. Iryna Pinchuk (Department of Surgery, UTMB). CCD841 and CT26 were purchased from ATCC within the past two years, and confirmed by ATCC. CT26 cells were previously termed MC-26 mouse colon cancer cells^55^. All cell lines were monitored regularly for absence of mycoplasma and HEK293 and HCT116 cell lines were confirmed to represent human epithelial cell lines with the help of Biosynthesis Company (Lewisville, TX). Stable clones of HEK293 cells were generated to overexpress either the control vector (HEKC) or a triple mutant hGAS vector, in order to overexpress full length progastrin (PG) peptide (HEKmGAS cells), as described previously^12^,^22^. The wild type parental cell lines (HEK293, HCT116) were cultured in DMEMF_12_ medium (Invitrogen, Grand Island, NY), supplemented with 10% FCS containing 1% penicillin/streptomycin in a humid atmosphere at 37 °C with 5% CO_2_. The stable clones of HEKC and HEKmGAS cells were cultured in the same medium supplemented with 100 μg/mL Geneticin (Invitrogen) under similar conditions. CCD841 and CT26 were similarly cultured using MEM (CCD841) and RPMI-1640 (CT26), media, along with supplements as described above. In addition, for screening purposes only, several panels of human colon cancer cell lines were purchased in January of 2015 from ATCC, and maintained in culture as suggested by the company.

### Procurement of samples from normal colonic mucosa and colonic tumors of patients

Samples of normal colonic mucosa were obtained from consented patients at the time of endoscopy for screening purposes, as per our approved IRB protocol (UTMB IRB #03-237). Normal samples were obtained only if the colons were free of adenomas (Ads) and adenocarcinomas (AdCAs), but positive for small hyperplastic (Hp) growths. Pinch biopsies of tubular adenomas (TAs) (polyps) were also obtained at the time of screening endoscopy, from patients who were positive for polyps but negative for AdCAs, as per our approved IRB Protocols; rest of the snared polyps were sent to pathology department. Samples of primary or metastatic tumors, with or without the adjoining uninvolved colonic tissue (matched paired sample) were obtained as discarded samples (as per our approved UTMB IRB protocol #91-310) from either UTMB Hospital, at time of surgery, or from Tissue Core Facility at Cancer Center, University of Alabama, as part of CHTN Program funded by NIH. All samples were collected and flash-frozen and stored in liquid nitrogen or −80 °C until analyzed. Pathology of all samples, thus obtained, was confirmed. In few experiments we also harvested tissue samples from colons, liver and brain of male FVB/N mice (2–4 month old) (Taconic, Hudson, NY) by our published methods^44^, as per our approved IACUC protocols (UTMB IACUC protocol #01-12-055). Ninety-two colorectal carcinoma tissues were used for clinical validation of DCLK1-S expression from an independent cohort, for data presented in Fig. 9 and Supplementary Tables 4 & 5. These specimens were preserved immediately after surgical resection in RNA later (QIAGEN, Chartsworth, CA) and stored at −80 °C until RNA extraction. The surgical samples were obtained from the Mie University Hospital, Japan, from patients enrolled during 2005 to 2011. The patients included 57 men and 35 women with a mean age of 68 years (range 37–89 years). None of the patients received chemotherapy and radiotherapy before surgery and no perioperative mortalities were observed. The primary lesion was located in the rectum in 41 patients, sigmoid colon in 19, ascending colon in 16, transverse colon in 9, and descending colon in 7. Eleven patients were diagnosed with synchronous liver metastasis. Clinicopathological findings were based on the UICC’s criteria for tumor node metastasis (TNM) classification. There were 19 patients with stage I (T1-2N0M0), 30 with stage II (T3-4N0M0), and 22 with stage III (TXN1-2M0) disease. Twenty-one patients with distant metastasis were classified as having stage IV (TXNXM1) disease. Ten patients had poorly differentiated or mucinous adenocarcinoma, whereas 82 patients had well or moderately differentiated colorectal tumors. Postoperative follow-up data were obtained from all patients, and the median follow-up duration was 21.8 months (range: 1–88). All patients were followed up after the initial hospital discharge, with physical examination and tumor marker assays (CEA, CA19-9) performed every 1–3 months and computed tomography performed every 6 months. Endoscopic examinations were performed when necessary. Written informed consent was obtained from each patient (as per approved BCM IRB protocol #005-134). **All tissues were collected in accordance with the approved guidelines set forth by UTMB and BCM for the IRB and IACUC protocols.**

### Analysis of tissue samples and cell lines by RT-PCR/qRT-PCR

Total RNA was isolated from cell lines in monolayer cultures at 60–70% confluency, or from human and mouse tissues (described above), using Trizol Reagent (Invitrogen), as previously described^55^,^56^. For qRT-PCR, the iTaq Universal SYBR Green Supermix (Bio-Rad, CA) was used as per the manufacturer’s instructions. Expression levels of DCLK1-S in tissues for data presented in Fig. 9 were normalized against GAPDH using the 2-ΔCt method, as previously described^57^. The primer sequences used for PCR amplification of cDNA for both RT-PCR/qRT-PCR amplification of the long (L) and short (S) isoforms of DCLK1 from either human (h) or mouse (m) specimens are provided in Supplementary Table 1. Electrophoresis gels presented were cropped to present all the bands observed within the range covered by the molecular markers used (between 100 bp and 1000 bp for RT-PCR data), in order to avoid primer-dimers seen towards the end of the run. Processing of the electrophoresis blots was applied equally across the entire image. Touch-up tools were not used to manipulate data. Relative band-density of electrophoresis blots was analyzed using Image J program (rsbweb.nih.gov/ij/download) and expressed as a ratio or % of β-actin in the corresponding samples.

### 3′–5′ Primer-extension-assay

Total RNA was extracted from HCT116 and HEK-293 cells as described above. Nascent RNA was isolated using a Click-iT Nascent RNA Capture Kit (Life Technologies) according to the manufacturer’s instructions. 5 ug of total RNA or nascent RNA was reverse transcribed using a DCLK1-common primer (primer 2 in Fig. 4a), that encompassed the nt sequence from homologous coding sequence of both long and short isoforms of DCLK1. The pool of cDNA was purified using a column (Oligo Clean & Concentrator, Zymogen). The purified cDNA was ligated to a non-mammalian adapter sequence (atgctgaaacgcgagagaaaccgcgtatcaacccc) at the 5′-end by T4 DNA ligase followed by purification of the ligated cDNA product. 2 μl of the ligated product was PCR amplified using the forward adapter primer (primer 1) and reverse primer 2. Using these primers, the expected size for the DCLK1-S transcript is 498 bps (NM_001195415.1) and for the DCLK1-L transcript is 1300 bps (NM_004734.4) as shown in Fig. 4a.

### Treatment of colon cancer cells with 5-Azacytidine (de-methylating agent)

HCT116 cells were seeded in 100 mm dishes at a density of 5 × 10^6^ cells/dish, one day prior to drug treatment. The cells were treated with 10 μM 5‐aza‐2′‐deoxycytidine (5-Azacytidine) on days 2 and 5 of culture. The cells were harvested on day 6 of culture and total RNA isolated. RNA was processed for measuring relative levels of DCLK1-L/S by RT-PCR.

### Generation of *DCLK1* 5′(α)-promoter-reporter (luciferase) constructs

The long isoform (Isoform 1) of human DCLK1 is transcribed from 5′-promoter (NM_004794.4 in the NCBI data base). Based on the published promoter sequence (AL160392.12), several primer sets were designed to amplify three promoter segments of 0.5 to >2.0 Kb of the 5′-promoter from −100 through −2234 nts using genomic DNA from either normal colonic mucosa or HEK-293 cells, which gave identical results. The primers were synthesized with the restriction sites Xho1 at 5′-end and HindIII at 3′ end, in order to clone into PGL2 basic vector (as shown in Supplementary Table 1). The PCR products were purified using QIAquick PCR Purification kit (Qiagen, Valencia, CA), cloned into luciferase expression vector (PGL2 basic vector, Promega, WI) and amplified in DH5α competent cells (New England Biosciences, MD). Positive colonies were processed for purifying the promoter-reporter expression plasmids; control plasmids lacked the DCLK1 5′-promoter sequences. In initial experiments promoter-reporter plasmids were transfected into HEK-293/HEKmGAS and HCT116 cells, and the construct which demonstrated the maximum luciferase activity (−2234/−504 promoter-luciferase construct) (termed DCLK1-L-LUC), was used in all the studies presented in Fig. 5. For a control experiment, the two functional TCF/LEF binding sites in the DCLK1-L-Luc construct (−1904 and −1591) were disrupted. The −1904 TCF/LEF binding site was disrupted by insertion of a Not1 restriction site and the −1591 TCF/LEF binding site was disrupted by insertion of a SacII restriction site. Using the PGL2 luciferase expression vector, the DCLK1-L-Luc-F primer (as shown in Supplementary Table 1) and Not1-R primer (GCGGCCGCAGTGCTCTCACTAGAAATAGTT) were used to amplify a 5′ Xho1 and 3′ Not1 fragment. Not1-F primer (GCGGCCGCGATCAATATCTTAGTAATATAAAGGAAG) and SacII-R primer (CCGCGG AGTGCTCTCACTAGAAATAGTT) were used to amplify a 5′ Not1 and 3′ SacII fragment. SacII-F primer (CCGCGGTTGCTACTGAGAGAGTCAAACAC) and DCLK1-L-Luc-R primer (as shown in Supplementary Table 1) were used to amplify a 5′ SacII and 3′ HindIII fragment. The 3 fragments were then ligated together and cloned into the PGL2 luciferase expression vector as described above. The mutant plasmid was confirmed and reporter-promoter assays were conducted as described above.

### Generation of promoter-reporter constructs for IntronV-(β)promoter of *DCLK1*-gene

The short isoform of DCLK1 (isoform 2) (NM_001195415.1 in NCBI data base) is transcribed from a promoter within IntronV, as recently reported for neuronal cells^17^. Unlike the 5′-promoter, the IntronV-promoter has a consensus TATA binding site at −918nt (Fig. 7a), and promoter-reporter constructs surrounding the TATA box have been shown to be active in promoter-reporter assays^17^. Therefore, promoter fragments within IntronV (−2503/−771 and −1348/−771) were amplified using genomic DNA, described above, and cloned into PGL2 basic vector at XhoI and HindIII sites. The purified IntronV-promoter-reporter constructs, DCLK1-Luc-S1 (−2503/−771) and DCLK1-Luc-S2 (−1348/−771), were confirmed by DNA sequencing in the recombinant Core Facility at UTMB. Primer sequences used for PCR amplification of the promoter segments are listed in Supplementary Table 1.

### Promoter-Reporter assays

Cells were transiently transfected with the indicated promoter-reporter constructs using FuGENE6 for 24–48 h, as per manufacturer’s instructions; control cells were transfected with empty pGL2 vector, lacking promoter sequences. In some experiments promoter-reporter plasmids were used for measuring activation of β-catenin (TOPFlash wild type and FOPFlash mutant), obtained from Dr. Bert Vogelstein (John Hopkins, Baltimore, MD), as previously described^22^. Transfected cells were lysed in luciferase assay lysis buffer and luciferin was added according to instructions of the manufacturer (E2510, Promega WI). Luciferase activity was measured using a luminometer (Dynex Technologies, VA) after 10 sec of addition of substrate, as previously described^22^.

### Chromatin Immunoprecipitation Assays (ChIP)

For ChIP assays, cells in culture (60–70% confluent), were fixed in 1% formaldehyde to crosslink DNA to bound proteins, and reaction stopped by adding 0.125 M glycine. Cells were washed with cold PBS, pelleted at 4 °C in the presence of protease inhibitor cocktail (Sigma) and re-suspended in 600μl of ChIP sonication buffer (1% Triton X-100, 0.1% deoxycholate, 50 mM Tris-pH 8.1, 150 mM NaCl, 5 mM EDTA and protease inhibitors), followed by sonication and centrifugation of fragments (600–700 bp long) at 10,000 RPM. The crosslinked chromatin supernatant was immunopreciptated using target-specific antibody (2–5 μg purified IgG) at 4 °C, overnight. Control samples contained no antibody. For obtaining input levels of the corresponding proteins, equivalent numbers of cells were also processed for Western Immunoblot analysis. Protein A/G Sepharose beads, pre-absorbed by Herring sperm DNA (100μg/ml) was added to the chromatin-antibody complex and centrifuged to sediment the beads. The beads were washed with cold buffers, and DNA eluted from the beads with elution buffer (1% SDS, 0.1% NaHCO_3_, 0.01% mg/ml Herring Sperm DNA). DNA in the supernatant was precipitated using high-salt method as described by Ishizawa *et al*.^58^. The extracted DNA was purified using a kit from Zymogen (Catalog number D4060), and 2 μl of the purified DNA was used for PCR amplification of the immunoprecipitated DNA with specific primers designed around the transcription factor binding site of interest. The primer sequences used for this purpose are listed in Supplementary Table 1.

### DNA Methylation Analysis using the method of bisulfite conversion

Genomic DNA was purified from cell lines and colon tissues using a kit from Qiagen, and 2–5 μg of gDNA was used for methylation analysis. Methylation analysis was conducted as described by Clark *et al*.^59^. Briefly, DNA was treated with sodium hydroxide (3 M) for denaturation followed by bisulfite deamination using hydroquinone/sodium bisulfite treatment (16 mM hydroquinone, 4 M sodium bisulfite), overnight at 50 °C. The reaction mixture was desalted using Wizard DNA clean-up kit (Promega) and NaOH (3.0 M), followed by incubating at 37 °C for 20 min for alkali de-sulphonation reaction. The DNA was precipitated in the presence of 10 mg/ml glycogen as a carrier by the method used by Ishizawa *et al*.^48^. Bisulfite converted DNA (2μl) was amplified by PCR using bisulfite converted primers (primers used are listed in Supplementary Table 1). The PCR products were purified by a column (Wizard DNA clean-up kit, Promega) and cloned into a TA cloning vector (Sigma). Clones were confirmed by EcoR1 digestion and positive clones were sequenced using T7 primers in the recombinant DNA Core Facility at UTMB.

### Western Immunoblot (WB) analysis

Treated and control cells growing as mono-layer cultures, were harvested and processed for preparing cellular-lysates, followed by electrophoresis and transferred to PVDF-membranes as previously described^11^,^12^,^22^. Frozen tissue samples obtained from patients as described above were homogenized and processed for preparation of tissue lysates in RIPA buffer as described previously^11^,^12^,^22^. Samples containing 30–50 μg of proteins were subjected to electrophoresis and transferred to PVDF-membranes as previously described^11^,^12^,^22^. Blots were cut into horizontal strips containing target or loading-control proteins (β-actin), and processed for WB, as described previously^11^,^12^,^22^. Antigen-antibody complexes were detected with a chemiluminescence-reagent kit (Thermoscientific, IL or GE Healthcare, UK). Membrane-strips containing either target or loading control proteins were simultaneously exposed for equal time to autoradiographic films. Western blots presented were cropped to exclude bands beyond the range of the molecular markers, at the running end and at the loading end, as is customary, which helps to develop both weak and strong signals within the relevant range. Processing of films was applied equally across the entire image. Touch-up tools were not used to manipulate data. Relative band-density on scanned autoradiograms was analyzed using Image J program (rsbweb.nih.gov/ij/download) and expressed as a ratio or % of β-actin in the corresponding samples.

### Transient-transfection of cells with oligonucleotides

Cell lines, seeded in 96-well plates were transfected with 5 pmol of either target-specific or control-siRNA, as indicated, using Lipofectamine^TM^ 2000 (Invitrogen), as described^11^,^22^. Transfected cells were propagated in normal growth medium containing 10% FCS, and processed for WB analysis after 48 h of transfection for confirming down-regulation of the target transcription factor (β-catenin or NF-κBp65). In order to examine the role of the indicated transcription factors in modulating the transcriptional activation of the promoter-reporter constructs, cells in culture were pre-transfected with the indicated promoter-reporter constructs, followed by transient transfection with the indicated siRNA molecules, followed by processing the cells after 48 h of treatment for relative levels of luciferase, as described above.

### Statistical analysis

Data are presented as mean ± SEM of values obtained from indicated number of patient samples or experiments. To test for significant differences between means, nonparametric Mann Whitney test was employed using STAT view 4.1 (Abacus Concepts, Inc, Berkley, CA). Chi-square tests were used to analyze the relationship between DCLK1-S expression and clinicopathological factors. Overall survival curves were analyzed using Kaplan-Meier method, and comparisons were made using the log-rank test. The cut off threshold between high and low expression group for DCLK1-S transcript was defined by the median values of the gene’s expression in cancerous tissue. The cox proportional hazards regression model, using Medcalc version 12.3.0 was utilized to estimate univariate and multivariate hazard rations for prognosis. In addition to target mRNA expression, a list of clinical variables was considered for univariate and multivariate analysis to determine its impact on prognosis of patients with colorectal cancer: sex, age at diagnosis (continuous), pathological differentiation (differentiated or undifferentiated), tumor size (>41 mm median or <41 mm), lymph node metastasis (present or absent), and distant metastasis (presence or absence). All p values were two-sided and differences were considered to be statistically significant if <0.05.

## Additional Information

**How to cite this article**: R O’Connell, M. *et al*. Epigenetic changes and alternate promoter usage by human colon cancers for expressing DCLK1-isoforms: Clinical Implications. *Sci. Rep*. **5**, 14983; doi: 10.1038/srep14983 (2015).

## Supplementary Material

Supplementary Information
