# Supplementary material for: Epigenetic changes and alternate promoter usage by human colon cancers for expressing DCLK1-isoforms: Clinical Implications
Source: Sci Rep. 2015 Oct 8;5:14983. doi: 10.1038/srep14983 (PMC4597220; doi:10.1038/srep14983)
Supplement: Supplementary Information [file srep14983-s1.pdf]

Supplementary Information

Epigenetic changes and alternate promoter usage by human colon cancers for expressing DCLK1-isoforms: Clinical Implications

Malaney O'Connell*, Shubhashish Sarkar*, Gurinder Luthra, Yoshinaga Okugawa

Yuji Toiyama, Aakash Gajjar, Suimin Qiu, Ajay Goel, Pomila Singh**

*equal contribution, **corresponding author

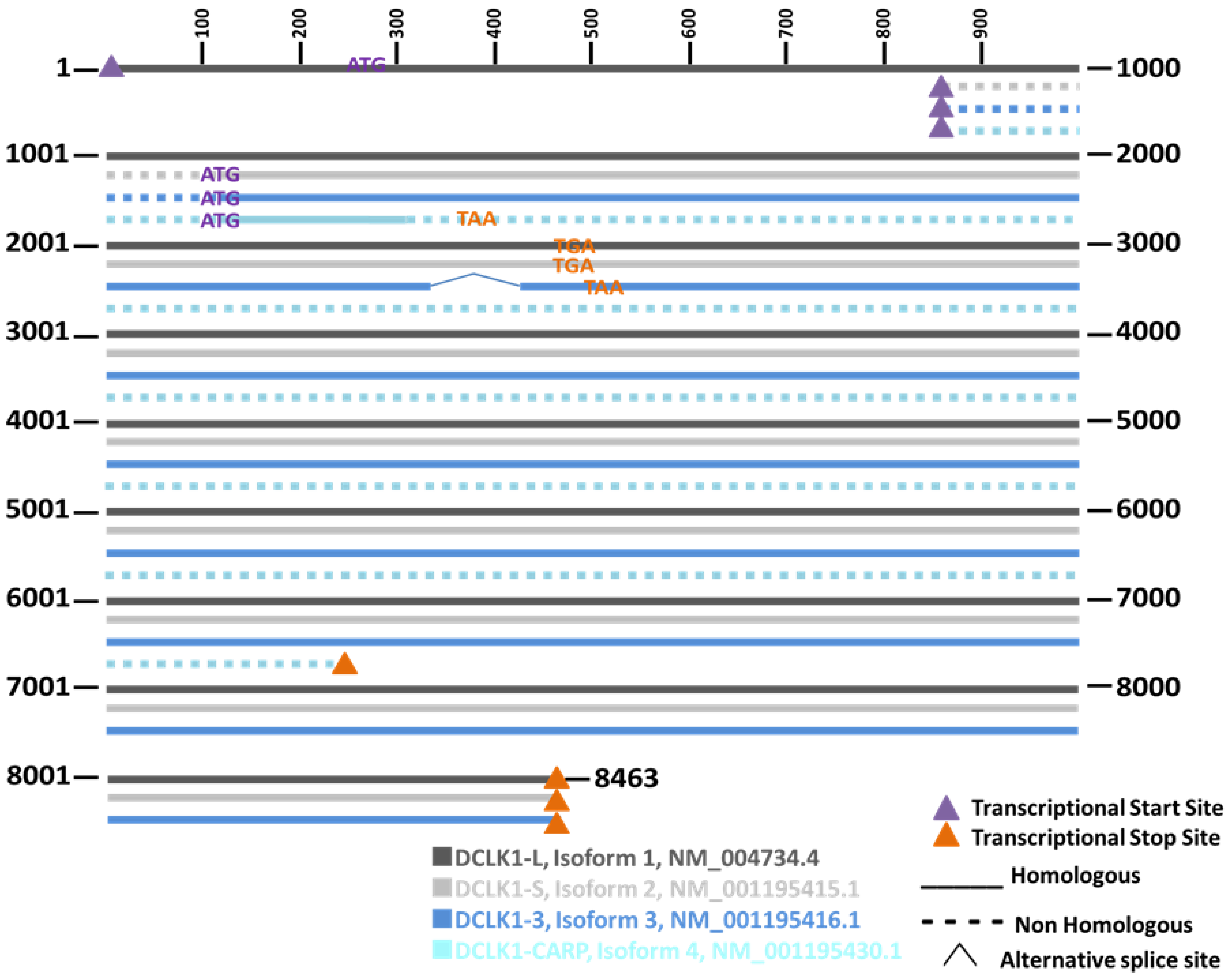

100
200
300
400
500
600
700
800
900
1
1000
ATG
1001
2000
ATG
ATG
ATG
TAA
2001
3000
TGA
TGA
TAA
3001
4000
4001
5000
5001
6000
6001
7000
7001
8000
8001
8463
Transcriptional Start Site
Transcriptional Stop Site
Homologous
Non Homologous
Alternative splice site
DCLK1-L, Isoform 1, NM_004734.4
DCLK1-S, Isoform 2, NM_001195415.1
DCLK1-3, Isoform 3, NM_001195416.1
DCLK1-CARP, Isoform 4, NM_001195430.1

SupplementaryFigure1. Nucleotide sequence homology between the transcripts for the 4 isoforms of human *DCLK1*. Diagrammatic representation of the nucleotide sequence homology of isoforms 1,2,3,4 of h*DCLK1* (formerly known as DCAMKL1), as described in the NCBI database. Dark Grey=isoform 1 (NM_004734.4), Light Grey=isoform 2 (NM_001195415.1), Dark Blue=isoform 3 (NM_001195416.1), and Light blue=isoform 4 (NM_001195430.1). Solid lines=homologous regions, dashed lines=non-homologous regions, arrow head=alternative splice site, purple triangles=transcriptional start sites, orange triangles=transcriptional stop sites. Start (purple) and stop (orange) codons are indicated. The coding region of *DCLK1* isoform 1 (arbitrarily termed long isoform, DCLK1-L) starts at bp 284 (from exon2, as shown in **Fig3a**) and ends at bp 2473 (exon18). The 5' untranslated region includes exon 1 and part of exon 2, downstream of 5'(α)-promoter as shown in **Figure3a**. The 3' untranslated region includes most of exon 18 (3301 bp). The coding region of *DCLK1* isoform 2 (arbitrarily termed short isoform, DCLK1-S) starts at base 334 (of exon 1, 3' of IntronV, as shown in **Fig3a**), and ends at base 1602 (in exon 14, as shown in **Fig3a**). Isoform 2 consists of 14 exons. The 5' untranslated region includes most of exon 1 (352 bp) and 3' untranslated region includes most of exon 14 (3301 bp). The coding region of *DCLK1* isoform 3 (DCLK1-3) starts at base 334 (in exon 1, 3' of IntronV) and ends at base 1635 (exon 14). Isoform 3 is transcribed from 13 exons rather than 14 exons. Isoforms 2 and 3 represent splice variants of transcripts originating in the same exon of the gene that are different by 74 bps, since isoform 2 contains sequences that are transcribed from an additional exon compared to isoform 3[SupplementaryReference1]. The coding region of *DCLK1* isoform 4 (DCLK1-Carp) starts at base 334 and ends at base 504, and is transcribed from 4 exons. The 5' untranslated region is homologous between isoforms 2, 3, and 4. The 3' untranslated region of isoform 4 includes most of exon 4. **Isoform 1 (DCLK1-L) and isoform 2 (DCLK1-S) share 7200 bps, and were the only two transcripts that were detected in normal colons and hcolon cancer cells.** The 5' UTR of L and S transcripts were non-homologous and at least 17bps of the S-transcript, from the ATG site, was also non-homologous; primers were thus designed from this non-homologous region to detect DCLK1-S transcripts, and the specific sense and anti-sense primers used for detecting either the L isoform or the S isoform are presented in **SupplementaryTable1**. *Sense and anti-sense primers were similarly designed from non-*

homologous sequences of the transcripts, to distinguish between isoforms 2, 3, and 4, and it was confirmed that normal and cancer cells examined in this study were positive for only isoforms 1 and 2, which have been arbitrarily termed long (L) and short (S) isoforms of DCLK1, for the purpose of these studies.

a

```
Long    1    MSFGRDMELEHFDERDKAQRYSRGSRVNGLPSPTHSAHCSFYRTRTLQTLSSEKKAKKVRFYRNGDRYFKGIVYAISPDR  80
Short        --------------------------------------------------------------------------------

Long   81    FRSFEALLADLTRTLSDNVNLPQGVRTIYTIDGLKKISSLDQLVEGESYVCGSIEPFKKLEYTKNVNPNWSVNVKTTSAS  160
Short        --------------------------------------------------------------------------------

Long  161    RAVSSLATAKGSPSEVRENKDFIRPKLVTIIRSGVKPRKAVRILLNKKTAHSFEQVLTDITDAIKLDSGVVKRLYTLDGK  240
Short        --------------------------------------------------------------------------------

Long  241    QVMCLQDFFGDDDIFIACGPEKFRYQDDFLLDESECRVVKSTSYTKIASSSRRSTTKSPGPSRRSKSPASTSSVNGTPGS  320
Short   1    -------------------------------------------------------------------MLELIEVNGTPGS  13

Long  321    QLSTPRSGKSPSPSPTSPGSLRKQRSSQHGGSSTSLASTKVCSSMDENDGPGEEVSEEGFQIPATITERYKVGRTIGDGN  400
Short  14    QLSTPRSGKSPSPSPTSPGSLRKQRSSQHGGSSTSLASTKVCSSMDENDGPGEEVSEEGFQIPATITERYKVGRTIGDGN  93

Long  401    FAVVKECVERSTAREYALKIIKKSKCRGKEHMIQNEVSILRRVKHPNIVLLIEEMDVPTELYLVMELVKGGDLFDAITST  480
Short  94    FAVVKECVERSTAREYALKIIKKSKCRGKEHMIQNEVSILRRVKHPNIVLLIEEMDVPTELYLVMELVKGGDLFDAITST  173

Long  481    NKYTERDASGMLYNLASAIKYLHSLNIVHRDIKPENLLVYEHQDGSKSLKLGDFGLATIVDGPLYTVCGTPTYVAPEIIA  560
Short 174    NKYTERDASGMLYNLASAIKYLHSLNIVHRDIKPENLLVYEHQDGSKSLKLGDFGLATIVDGPLYTVCGTPTYVAPEIIA  253

Long  561    ETGYGLKVDIWAAGVITYILLCGFPPFRGSGDDQEVLFDQILMGQVDFPSPYWDNVSDSAKELITMMLLVDVDQRFSAVQ  640
Short 254    ETGYGLKVDIWAAGVITYILLCGFPPFRGSGDDQEVLFDQILMGQVDFPSPYWDNVSDSAKELITMMLLVDVDQRFSAVQ  333

Long  641    VLEHPWVNDDGLPENEHQLSVAGKIKKHFNTGPKPNSTAAGVSVIALDHGFTIKRSGSLDYYQQPGMYWIRPPLLIRRGR  720
Short 334    VLEHPWVNDDGLPENEHQLSVAGKIKKHFN--------------------------------------------------  363

Long  721    FSDEDATRM  729
Short        ---------
```

b

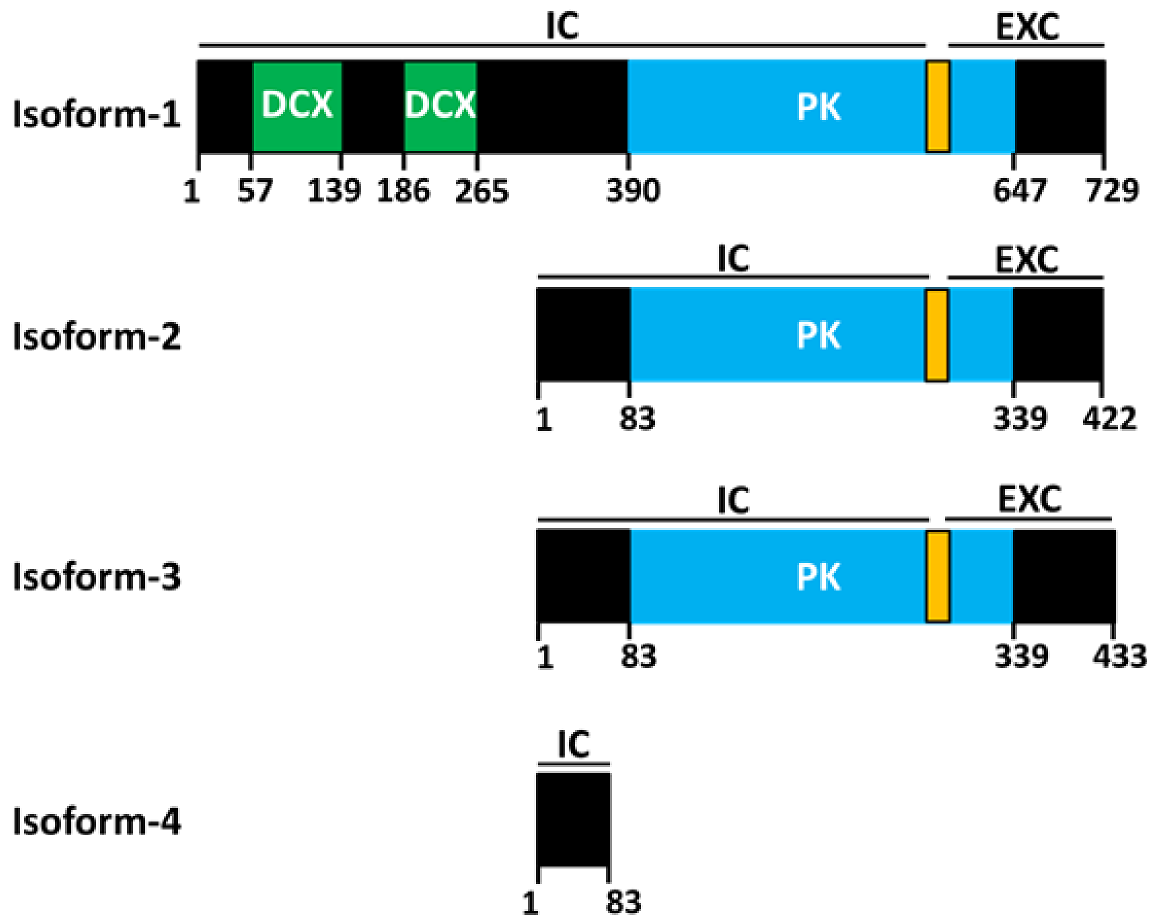

SupplementaryFigure2. (a) Homology between the amino-acid sequence of isoforms 1 (DCLK1-L) and 2 (DCLK1-S). Amino-acid sequences of the two L/S isoforms have been aligned, and homologous sequences are underlined. Only a few amino acids in the S isoform were different and have been boxed. Dashed line depicts absence of corresponding amino acids in S isoform compared to the L isoform. (b) diagrammatic representation of protein domains in the 4 isoforms of hDCLK1. DCX=doublecortin domain; PK=calmodulin-like proten kinase domain; IC=intracellular domains of the indicated isoforms; EC=extracellular domain of the four isoforms; **Isoforms are named according to the NCBI database.** The total number of amino acids in the four isoforms, as it relates to the various domains is indicated. Briefly, isoform (DCLK1-L) consists of two doublecortin domains, a protein kinase domain, and a serine/proline rich area. The transmembrane domain was present only in isoforms 1, 2 and 3. Protein analysis using PSORT (www.psort.nibb.ac.jp.org), University of Tokyo, Japan) suggested only one transmembrane domain (located between 568-584 AAs of isoform 1); labeled as DCLK1-L in the current studies). However, protein analysis using BCM Search Launcher (http://searchlauncher.bcm.tmc.edu/multi-align/multi-align.html), Baylor College of Medicine, Houston, TX) identified 2 transmembrane domains (located between AA 534-559 and 568-585). Isoforms 2 and 3 retain the protein kinase domain but lack the doublecortin domains, compared to isoform 1. Isoform 4 lacks both the doublecortin domains and the protein kinase domain. *It is important to note that SwissProt describes a 740 AA isoform (015075-1) that is not described in the NCBI database.* This 740 AA protein consist of two doublecortin domains, a protein kinase domain, and a serine/proline rich area. **Various names have been given to the different isoforms in literature**. Isoform 1 (NP_004725.1) has been previously referred to as DCLK1-long-B[26], KIAA0369AS[27], DCK-α1[31], and DCLK1 β[29]. Isoform 2 (NP_001182344.1) has been previously referred to as DCLK1-short-B[26], DCK-β1[28], and KIAA0369-BS[27]. Isoform 3 (NP_001182345.1) has been previously referred to as DCLK1-short-A[26], DCK-β2[28], KIAA0369-BL[27], and CPG16[29,30]. Isoform 4 has been previously referred to as ania-4[SupplementaryReference2] and CARP[SupplementaryReference3]. The 740 AA protein described in the SwissProt database (015075-1) has been referred to as DCLK1-long-A[26], KIAA0369-AL[27], DCK-α2[28], and DCLK α[29]. Most investigators in this field have been using commercially available antibodies

which were generated against a peptide sequence between 700-729 bp of isoform 1 (DCLK1-L), which is homologous to C-terminal end of isoform 2 (DCLK1-S), but is not homologous to isoforms 3 and 4. Since we now know the normal and cancer epithelial cells, examined in here, only express isoforms 1 (L) and 2 (S), the commercially available antibodies detect both these isoforms, which differ in molecular mass: L= ~82 kDa; S= ~47kDa.

a

	CpG Sites(● Methylated: ○ Unmethylated)																			
**Normal**	**1**	**2**	**3**	**4**	**5**	**6**	**7**	**8**	**9**	**10**	**11**	**12**	**13**	**14**	**15**	**16**	**17**	**18**	**19**	**20**
1	○	○	●	○	●	○	○	○	○	○	○	○	○	○	○	○	○	●	○	○
2	○	○	●	○	●	○	○	○	●	●	○	○	○	○	○	○	○	●	●	●
3	○	○	○	○	●	●	○	●	●	○	○	○	○	○	○	○	○	○	○	●

	CpG Sites (● Methylated:○ Unmethylated)																			
**TA**	**1**	**2**	**3**	**4**	**5**	**6**	**7**	**8**	**9**	**10**	**11**	**12**	**13**	**14**	**15**	**16**	**17**	**18**	**19**	**20**
1	○	●	●	●	○	●	○	●	○	●	●	○	○	●	●	○	●	●	○	●
2	○	●	○	○	●	○	●	○	●	○	○	○	○	●	●	●	○	●	●	●
3	○	●	●	●	○	●	○	●	●	●	●	●	●	●	●	○	●	●	●	●

	CpG Sites (● Methylated:○ Unmethylated)																			
**Primary**	**1**	**2**	**3**	**4**	**5**	**6**	**7**	**8**	**9**	**10**	**11**	**12**	**13**	**14**	**15**	**16**	**17**	**18**	**19**	**20**
1	●	●	●	●	●	●	●	●	●	●	●	●	●	●	○	●	●	●	●	●
2	○	●	●	●	○	●	○	●	●	●	●	●	●	●	●	●	●	●	●	●
3	○	●	●	●	●	●	○	●	●	●	●	●	●	●	●	○	●	●	●	●

	CpG Sites (● Methylated:○ Unmethylated)																			
**METS**	**1**	**2**	**3**	**4**	**5**	**6**	**7**	**8**	**9**	**10**	**11**	**12**	**13**	**14**	**15**	**16**	**17**	**18**	**19**	**20**
1	○	●	●	●	○	●	○	●	●	●	●	●	●	●	●	○	●	●	●	●
2	○	●	●	●	○	●	○	●	●	●	●	●	●	●	●	○	●	●	●	●
3	○	●	●	●	●	○	●	○	●	●	●	●	●	●	●	●	○	●	●	●

SupplementaryFigure3. DNA methylation analysis of 5'(α)-promoter of h*DCLK1* in human samples. Methylation status of all twenty CpG sites in representative normal, tubular adenoma (TA), primary tumors (primary), and metastatic tumors (METS) is presented. Open circles=unmethylated CpG sites; filled circles=methylated CpG sites. The procurement of all the samples has been described under Methods.

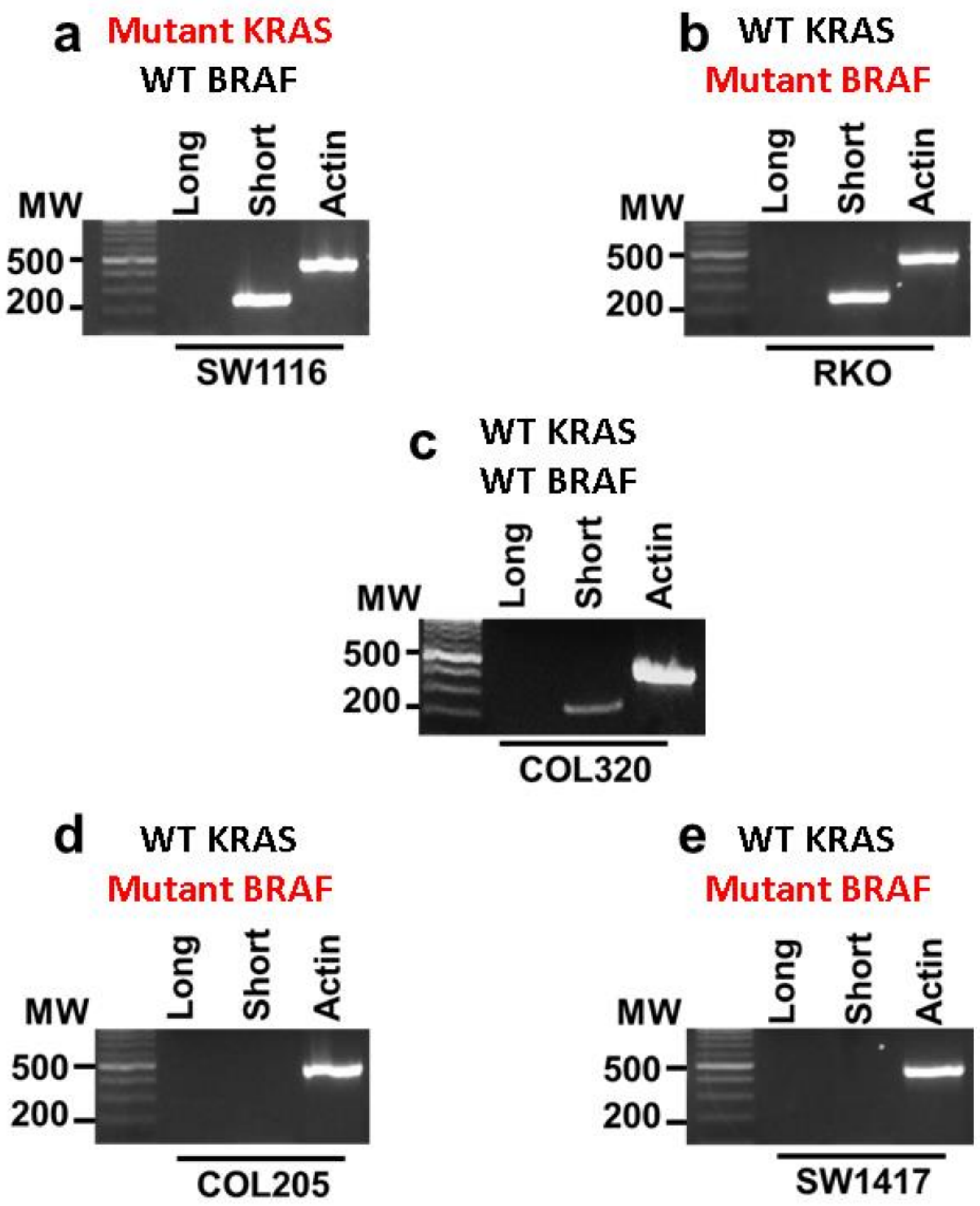

SupplementaryFigure4. Representative RT-PCR analysis of long and short transcripts of DCLK1 in human colon cancer (hCCC) cell lines. Representative RT-PCR data are shown from: hCCC cell lines (a-e); β-actin was run as internal controls. The molecular weight (MW) in terms of bps is shown on left-hand side of each image.

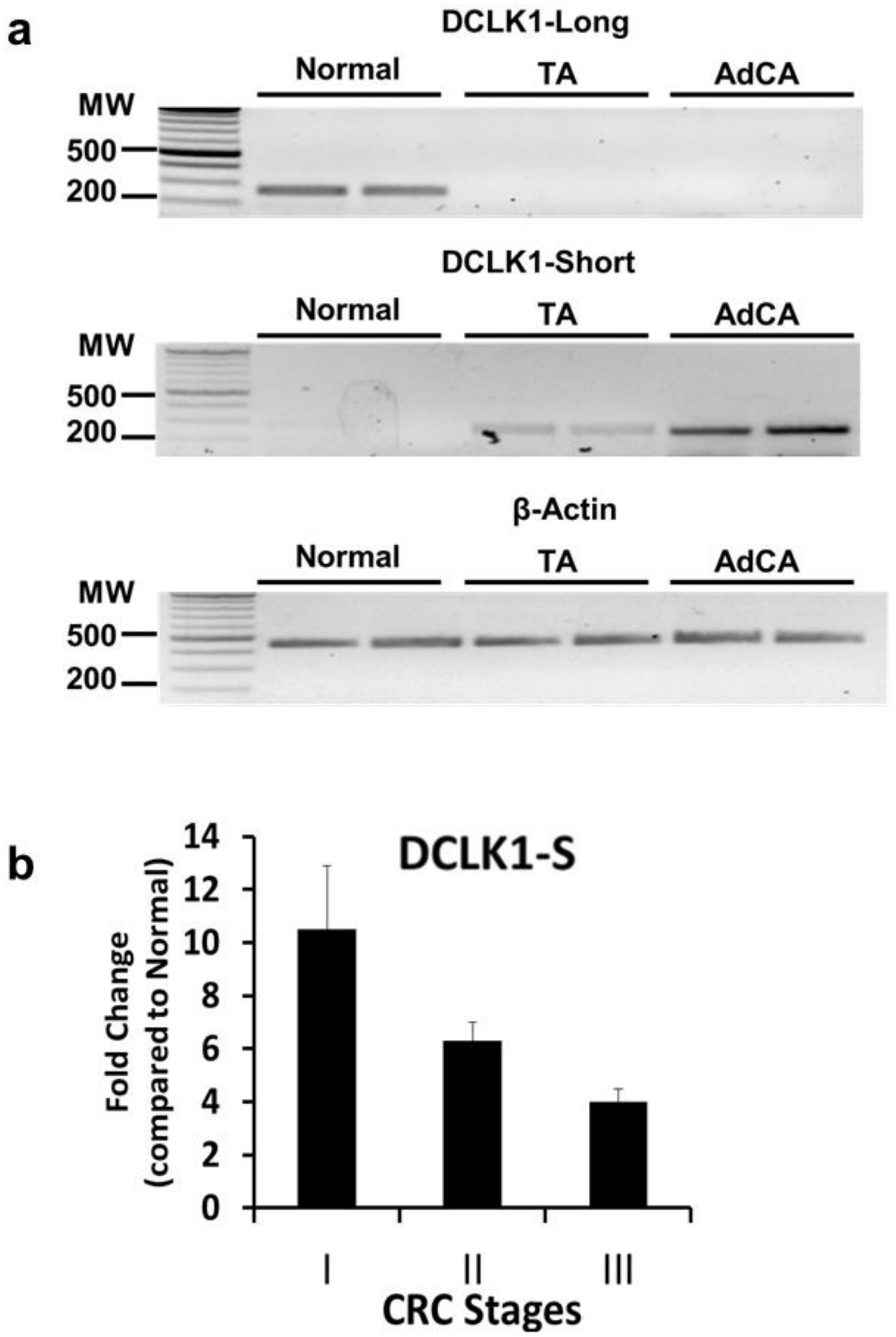
a
DCLK1-Long
Normal
TA
AdCA
MW
500
200
DCLK1-Short
Normal
TA
AdCA
MW
500
200
β-Actin
Normal
TA
AdCA
MW
500
200
b
DCLK1-S
Fold Change (compared to Normal)
14
12
10
8
6
4
2
0
I
II
III
CRC Stages

SupplementaryFigure5. Relative expression levels of long and short transcripts of DCLK1-isoforms in patient samples. Total RNA from indicated tissue samples were amplified by RT-PCR by using sense and anti-sense primers for amplifying either DCLK1-L transcripts or DCLK1-S transcripts, as described in Methods. Representative data are presented from normal colons, tubular adenomas (TA) and advanced adenocarcinomas (AdCAs), obtained from two patients each; the samples were co-amplified and co-run at the same time for L/S transcripts and corresponding β-actin levels. Densitometric data from all samples that were similarly analyzed are presented as a ratio of β-actin levels in the corresponding samples, and are presented as bar-graphs in **Figure3g,h**.

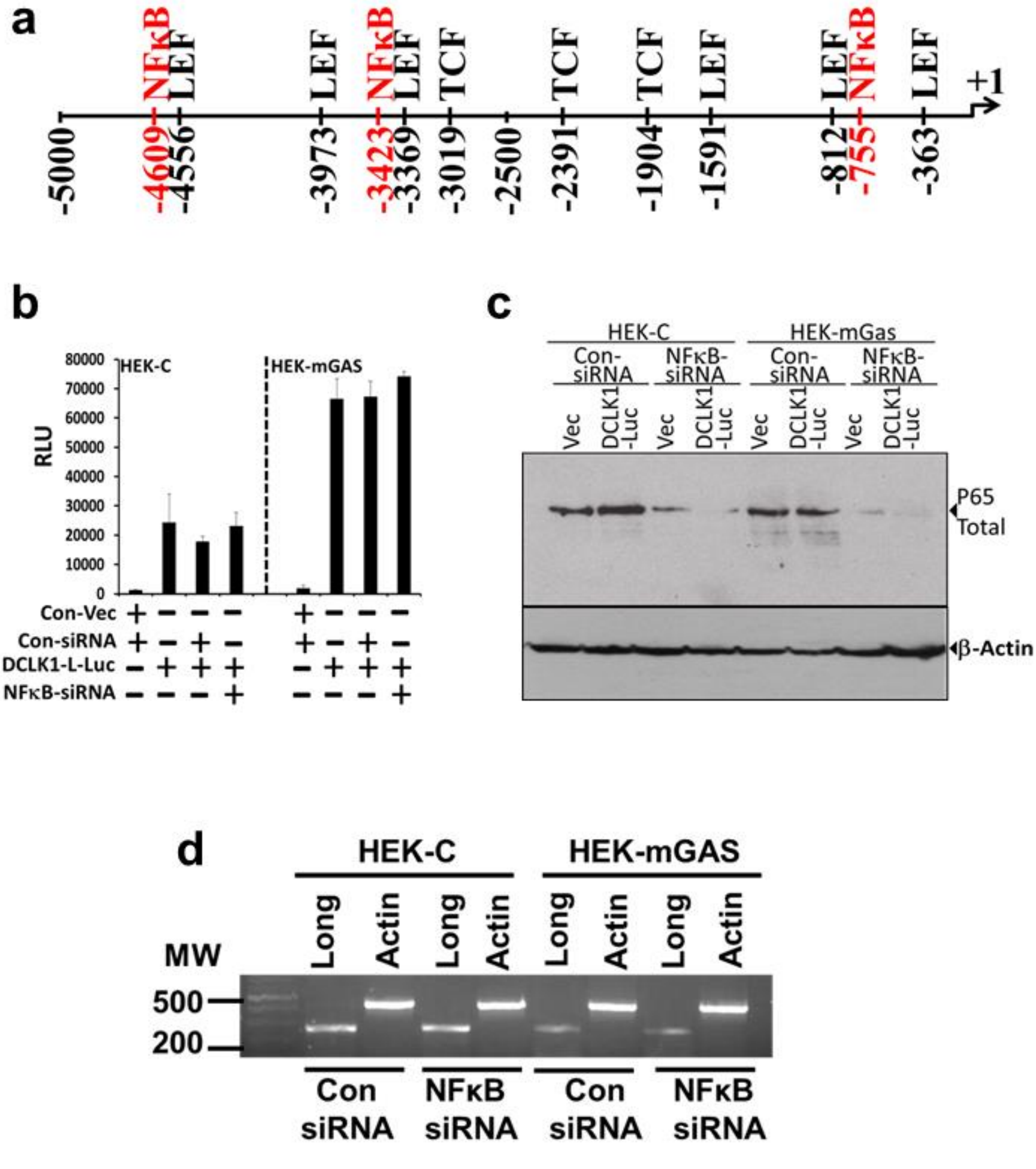
a
NFκB LEF LEF NFκB LEF TCF TCF TCF LEF LEF NFκB LEF
+1
-5000 -4609 -4556 -3973 -3423 -3369 -3019 -2500 -2391 -1904 -1591 -812 -755 -363
b
HEK-C
HEK-mGAS
RLU
80000 70000 60000 50000 40000 30000 20000 10000 0
Con-Vec
Con-siRNA
DCLK1-L-Luc
NFκB-siRNA
c
HEK-C
HEK-mGas
Con-siRNA
NFκB-siRNA
Vec
DCLK1-Luc
P65 Total
β-Actin
d
HEK-C
HEK-mGAS
Long
Actin
MW
500
200
Con siRNA
NFκB siRNA

SupplementaryFigure6. Role of NFκB binding site in activation of the 5'(α)-promoter of DCLK1. **(a)** In silico analysis of ~5kb of 5'-promoter of human *DCLK1*-gene (transcribing DCLK1-L), identified several binding sites for TCF-4/LEF and three NFκB binding sites, with >90% conserved sequences. (**b)** = Relative transcriptional/luciferase activity (RLU) in the indicated cells, transiently-transfected with the plasmids for 48h, in the presence or absence of transfection with either control or NFκBp65-siRNA. Cells were co-transfected with promoter-reporter construct±siRNA. VEC=control LUC vector. Each bar represents mean±SEM of four experiments. (**c) =** Western-Blot analysis, demonstrating efficacy of NF-κBp65-siRNA for down-regulating the expression of NF-κBp65 protein in the cell lines. (**d) =** Representative RT-PCR data for DCLK1-L isoform from indicated cells, in the presence of absence of either control-siRNA or NFκBp65-siRNA. The indicated cell lines were transfected with either (scrambled) siRNA (con-siRNA) or target-specific NF-κBp65-siRNA for 48h, before processing the cells by RT-PCR.

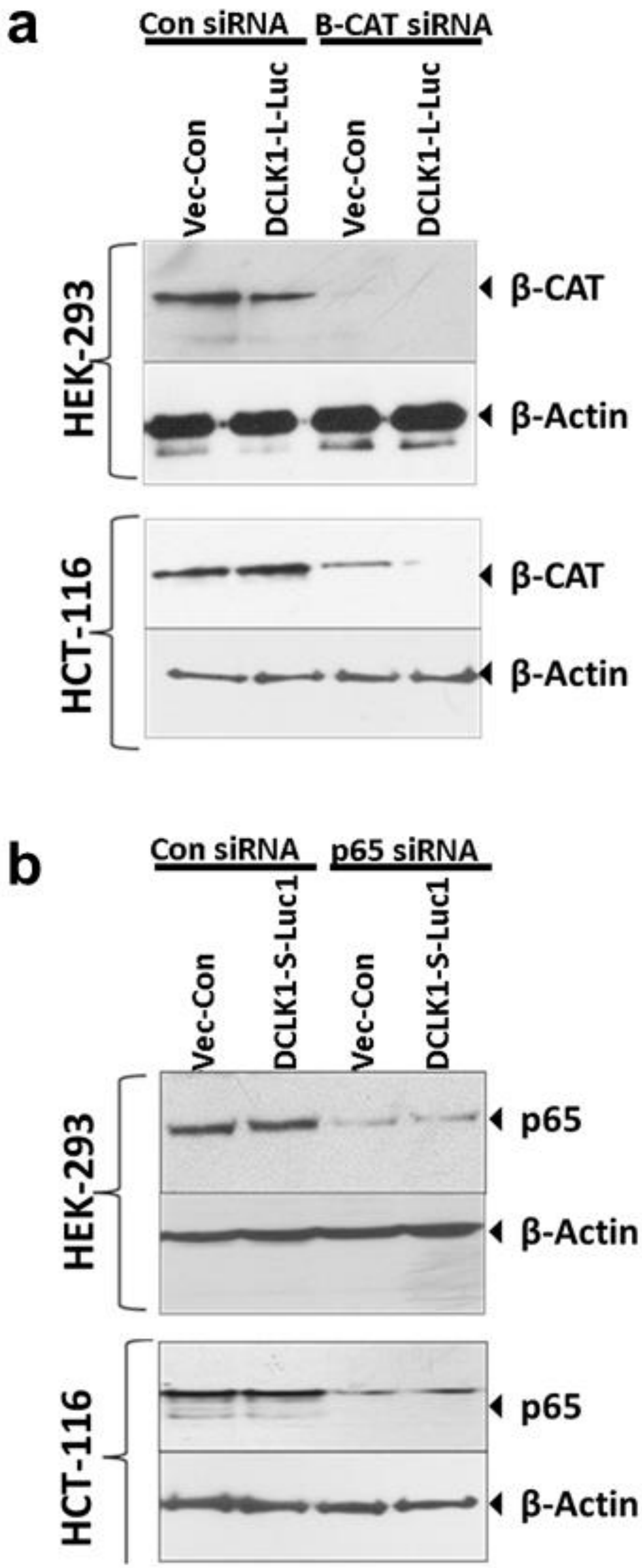
a
Con siRNA
B-CAT siRNA
Vec-Con
DCLK1-L-Luc
Vec-Con
DCLK1-L-Luc
HEK-293
β-CAT
β-Actin
HCT-116
β-CAT
β-Actin
b
Con siRNA
p65 siRNA
Vec-Con
DCLK1-S-Luc1
Vec-Con
DCLK1-S-Luc1
HEK-293
p65
β-Actin
HCT-116
p65
β-Actin

SupplementaryFigure7. Western-Blot analysis, demonstrating efficacy of β-catenin-siRNA and NF-κBp65-siRNA for down regulating the expression of the corresponding protein in the cell lines. The indicated cell lines were transfected with either empty vectors (Vec-con) or vectors expressing promoter-reporter constructs (DCLK1-L-LUC) **(a)**, or DCLK1-S-LUC-1) **(b)**, and co-transfected with either control (scrambled) siRNA (con siRNA) or target-specific siRNA: β-catenin-siRNA **(a)** or NF-κBp65-siRNA **(b)**, for 48h, as described in Methods. In each case the target specific siRNA was effective in significantly down-regulating the expression of the target proteins by >80-90%. The data presented are representative of three separate experiments conducted similarly in duplicate. β-cat=β-catenin; p65=NF-κBp65; Vec-con=empty vector; Con=control. The corresponding β-actin levels in each sample are shown.

Supplementary Table 1<br>Primer Sequences Used for RT-PCR/qRT-PCR And Other Assays			
**Target cDNA/gDNA**	**Species**	**Primer Sequence**	**Assay**
DCLK1-Long (cDNA)	Human	**F:**GGAGTGGTGAAACGCCTGTAC<br>**R:**GGTTCCATTAACTGAGCTGG	RT-PCR & qRT-PCR
DCLK1-Short (cDNA)	Human	**F:**ACACTAAGACTGTGTCCATGTTAGAACTC<br>**R:**AAGCCTTCCTCCGACACTTCT	RT-PCR & qRT-PCR
DCLK1-Long (cDNA)	Mouse	**F:**TCAATGAGGACCAGCTCCAG<br>**R:**TCCGAGAGAGTTCGGGTCA	RT-PCR & qRT-PCR
DCLK1-Short (cDNA)	Mouse	**F:**AAGACGTCAGCCTTACGCAG<br>**R:**GAGAGATCCTCTGCTTCCGC	RT-PCR & qRT-PCR
-1443 TCF *cis* element in 5' promoter (gDNA)	Human	**F:**AGAGCTGTGTCTGCTTGG<br>**R:**GTTCATTCCAGGGCAGCTTA	ChIP PCR
-1141 TCF *cis* element in 5' promoter (gDNA)	Human	**F:**TAAGCTGCCCTGGAATGAAC<br>**R:**CCCAAGCTATGCACTCTGGT	ChIP PCR
NF-κB *cis* element in intron V promoter (gDNA)	Human	**F:**CTGTATCCACTGCCCTCTGT<br>**R:**GCAAAGCTATCTTCAGGAGG	ChIP PCR
DCLK1-5' promoter(-1067/-650) (gDNA)	Human	**F:**TTTAGGGGTGTAGTTAAGTTAGATG<br>**R:**AACCTCTCTCTCCAAAAAAAAA	DNA Methylation of CpG sites
DCLK1-L-Luc (-2234/-503) (gDNA)	Human	**F:**ACATGACTGTGGGCAAATGA<br>**R:**CCCAAGCTATGCACTCTGGT	Promoter Reporter Construct
DCLK1-S-Luc1(-2503/-771)	Human	**F:**GGTGCTTCCGTTCAAAGTGT<br>**R:**CAGTCTCAGGAATACCTTGC	Promoter Reporter Construct
DCLK1-S-Luc2(-1348/-771)	Human	**F:**CCTCCTGAAGATAGCTTTGC<br>**R:**CAGTCTCAGGAATACCTTGC	Promoter Reporter Construct
Primer 1-Adaptor	Non-Human	**F:**GAGAACCGCGTATCAACCCC	LM-PCR
Primer 2-DCLK1 common	Human	**R:**GTGACGTAGAGGAGCCGCCA	LM-PCR

SupplementaryTable1. Oligonucleotide (primer) sequences used for qRT-PCR/RT-PCR/ChIP/promoter-methylation assays. The forward (F) and reverse (R) primer sequences that were designed and used for amplifying the indicated target cDNA/gDNA for conducting the different assays are shown.

Supplementary Table 2	
Relative Expression Of DCLK1-L/S In Normal Colonic Mucosa Samples From a total of22 Patients By Western Blot Analysis	
**DCLK1-L/S Expression**	**# of Patients**
**DCLK1-L High Expression**	**16/22**
**DCLK1-L Low Expression**	**5/22**
**DCLK1-L No Expression**	**1/22**
**DCLK1-S High Expression**	**1/22**
**DCLK1-S Low Expression**	**6/22**
**DCLK1-S No Expression**	**15/22**

SupplementaryTable2. Relative Expression Of DCLK1-L/S In Normal Colonic Mucosa Samples From 22 Patients By Western Blot Analysis. Normal colonic mucosa samples were collected from a total of 22 patients, free of adenocarcinomas, as described under methods. Relative band density in the western blots of normal colonic mucosa samples was analyzed using Image J, as described in Methods. Samples which expressed similar concentrations as HEK293 cells, used as positive control, were arbitrarily grouped under DCLK1-L high expression; samples which expressed less than 50% of that in HEK293 cells were arbitrarily grouped as DCLK1-L low expression; samples with no detectable expression, similar to that in HCT116 cells were labeled DCLK1-L No expression. Levels of DCLK1-S expression were quantified similarly, but HCT116 cells were used as a positive control, and HEK293 ce4lls were used as a negative control. Only one normal colon mucosal sample from patient 262, appeared to be negative for both S/L isoforms, which could be due to possible degradation of the sample, since samples after endoscopic collection, are usually flash frozen within 5min, but due to logistics can remain at room temperature for longer than 10 min, before flash freezing, as described.

Supplementary Table 3

RT-PCR analysis of long and short transcript of DCLK1 in human colon cancer cell lines

Cell Line	ATCC #	DCLK1 LONG	DCLK1 SHORT	Mutant Gene(s)
LOVO	CCL-229	-	+	APC, KRAS^{13}, MSH2
SW1116	CCL-233	-	+	APC, KRAS^{12}, TP53
SW837	CCL-235	-	+	APC, KRAS^{12}, TP53
SW948	CCL-237	-	+	APC, APC, KRAS^{61}, PIK3CA
HCT116	CCL-247	-	+	KRAS^{13}, PIK3CA
SW-480	CCL-228	-	+	APC, KRAS^{12}, SMAD4
DLD1	CCL-221	-	+	APC, KRAS^{13}, PIK3CA, TP53
COLO205	CCL-222	-		APC, BRAF, SMAD4, TP53
RKO	CRL-2577	-	+	BRAF, PIK3CA
LS411N	CRL2159	-	+	APC, BRAF, TP53
SW1417	CCL-238	-		APC, BRAF, PIK3R1, TP53
HT29	HTB-38	-	+	APC, BRAF, PIK3CA, SMAD4, TP53
NCIH508	CCL-253	-	+	BRAF, PIK3CA, TP53
Caco2	HTB-37	-	+	APC, SMAD4, TP53
COLO320	CCL-320	-	+	APC, TP53

SupplementaryTable3. RT-PCR analysis of long and short transcript of DCLK1 in human colon cancer cell lines. DCLK1-L and S primers were used to identify the isoforms being expressed by 15 colon cancer cell lines. The cell line name, ATCC catalog number, and mutational status of each cell line is provided. Most of these cell lines were purchased from ATCC in January of 2015. Cells positive for either DCLK1-L or S are represented by + sign, while cells negative for DCLK1-L or S are represented by - sign.

Supplementary Table 4					
**Clinicopathological Variables and DCLK1-S Expression In 92 Patients with CRCs**					
**Variable**		**n**	**High (n=46)**	**Low (n=46)**	**P Value**
Gender	Male	57	29	28	1.000
	Female	35	17	18	
Age (years)	<68 (median)	47	20	27	0.211
	≧68	45	26	19	
Tumor Size	≧4.1cm (median)	47	22	25	0.677
	< 4.1cm	45	24	21	
Histological Type	Differentiated	82	40	42	0.738
	Undifferentiated	10	6	4	
Pathological T Category	pT1	11	3	8	**0.019***
	pT2	12	4	8	
	pT3	59	32	27	
	pT4	10	7	3	
Vessel Involvment	Positive	42	25	17	0.143
	Negative	50	21	29	
Lymphatic Vessel Involvement	Positive	70	41	29	**0.007***
	Negative	22	5	17	
Lymph Node Metastasis	N0	51	21	30	0.093
	N1	41	25	16	
Distant Metastasis	M0	71	35	36	1.000
	M1	21	11	10	
TNM Stage	Stage I	19	5	14	0.061
	Stage II	30	15	15	
	Stage III	22	15	7	
	Stage IV	21	11	10	
**#Pearson's chi-square-test; *p<0.05**					

SupplementaryTable4. Clinicopathological variables and DCLK1-S expression in 92 colorectal cancer patients. Samples were obtained from patients with colonic adenocarcinomas at CRC stages of I-IV, from 92 patients in Japan, as described in Methods. The relative expression levels of DCLK1-S were analyzed by qRT-PCR, and high/low expression groups were classified by the median expression values in cancer tissues.

Supplementary Table 5

Multivariate Analysis for Predictors of Overall Survival

**Variables**	**Univariate**			**Multivariate**		
	HR	***95%Cl***	***p value***	***HR***	***95%Cl***	***p value***
Gender (Male vs. Female)	1.2	0.52-2.77	0.66	1.53	0.57-4.13	0.41
Age (≧68 (median) vs. <68)	1.26	0.58-2.75	0.56	0.77	0.29-2.05	0.6
Histological type (Undifferentiated/differentiated)	3.49	1.39-8.77	**0.008***	4.46	1.54-12.9	**0.006***
Tumor Size (≧4.1cm (median) vs. <4.1cm)	1.33	0.62-2.87	0.47	1.22	0.48-3.11	0.68
Lymph Node metastasis(present/absent)	13	3.90-43.1	**<0.001***	4.70	1.17-18.8	**0.03***
Distant metastasis(present/ absent)	9.67	4.35-21.5	**<0.001***	11.2	3.65-34.6	**<0.001***
DCLK1-S expression(high/low)	3.55	1.41-8.99	**0.008***	7.93	2.25-27.9	**0.0014***
	**HR=hazard ratio**		**Cl=confidence level**		***p<0.05**	

SupplementaryTable5. Multivariate Analysis for Predictors of Overall Survival. Cox's proportional hazards models were used to estimate hazard ratios (HRs) for overall survival. In multivariate analysis, undifferentiated histological type, lymph node metastasis, distant metastasis, and high DCLK1-S expression were independent prognostic factors in the cohort of 92 CRC patients. Cl=confidence level; *p<0.05 for the indicated variables.

Supplementary References

1. Sossey-Alaoui, K. & Srivastava, A. K. DCAMKL1, a brain-specific transmembrane protein on 13q12.3 that is similar to doublecortin (DCX). *Genomics* **56**, 121-126, doi:10.1006/geno.1998.5718 (1999).
2. Berke, J. D., Paletzki, R. F., Aronson, G. J., Hyman, S. E. & Gerfen, C. R. A complex program of striatal gene expression induced by dopaminergic stimulation. *J Neurosci* **18**, 5301-5310 (1998).
3. Vreugdenhil, E. et al. Kainate-elicited seizures induce mRNA encoding a CaMK-related peptide: a putative modulator of kinase activity in rat hippocampus. *J Neurobiol* **39**, 41-50 (1999).
